# Gut microbiome modulation by Weifuchun Capsules alleviates chronic atrophic gastritis: a combined microbiota and metabolomics approach

**DOI:** 10.3389/fmicb.2025.1634410

**Published:** 2025-09-02

**Authors:** Weiwei Xie, Nan Wang, Ming Wang, Qian Zhang, Wenyu Li, Huiyi Zhang, Yiran Jin, Yingfeng Du

**Affiliations:** ^1^Department of Clinical Pharmacy, The Second Hospital of Hebei Medical University, Shijiazhuang, Hebei, China; ^2^Department of Pharmaceutical Analysis, School of Pharmacy, Hebei Medical University, Shijiazhuang, Hebei, China

**Keywords:** chronic atrophic gastritis, gut microbiota, network pharmacology, untargeted metabolomics, Weifuchun Capsules

## Abstract

**Background:**

Weifuchun Capsule (WFC), a traditional Chinese medicinal formula containing *Panax ginseng* (red ginseng), *Rabdosia rubescens*, and *Fructus Aurantii*, is widely used clinically for the management of chronic atrophic gastritis (CAG). Although previous clinical evidence has demonstrated the efficacy of WFC in alleviating symptoms and improving gastric mucosal health, the precise mechanisms, particularly those mediated by gut microbiota, remain poorly understood. Given the crucial role of intestinal microbial dysbiosis in gastrointestinal disorders, exploring the microbiota-dependent pharmacological mechanisms of WFC is essential for understanding its therapeutic benefits in CAG.

**Methods:**

We established a rat model of CAG to investigate the microbiota-associated mechanisms underlying the effects of WFC treatment. Integrated microbiome–metabolome analyses were performed, combining 16S rRNA gene sequencing for gut microbiota profiling and untargeted metabolomics to detect shifts in metabolic pathways. Network pharmacology identified bioactive compounds from 99 characterized components in WFC, with molecular docking analyses further validating these findings. Correlations between gut microbial composition and metabolic profiles were assessed using Spearman’s analysis, and western blotting was employed to evaluate inflammation-associated proteins.

**Results:**

Network pharmacology screening identified 10 bioactive components from the 99 constituents present in WFC. Treatment with WFC significantly restored gut microbiota diversity and composition in CAG rats, notably enriching four bacterial families and ten genera. Metabolomic profiling revealed substantial regulation of glycerophospholipid and arachidonic acid metabolism, pathways intricately linked to microbial activity and gastrointestinal inflammation.

**Conclusion:**

Our findings highlight that modulation of gut microbiota composition is central to the therapeutic effects of WFC on CAG. WFC exerts its gastroprotective activity primarily by reshaping specific gut microbial populations and subsequently normalizing associated metabolic pathways. This microbiome-oriented perspective provides new insights into traditional herbal medicine mechanisms, emphasizing the critical need to understand microbiota-mediated therapeutic strategies in gastrointestinal disorders.

## 1 Introduction

Gastric cancer ranks among the most common malignancies and is the fourth leading cause of cancer-related death globally (Sung et al., 2021; Guan et al., 2023). Due to its asymptomatic or mild presentation in early stages, timely detection remains difficult (Li et al., 2022a). Gastric carcinogenesis typically progresses along the Correa cascade, with chronic atrophic gastritis (CAG) serving as a critical precancerous stage (Correa, 1992), underscoring the importance of early intervention.

Weifuchun Capsule (WFC), a standardized traditional Chinese patent medicine listed in the Chinese Pharmacopeia, comprises *Panax ginseng* C.A.Mey. (red ginseng, RG), *Isodon amethystoides* (Benth.) H.Hara (IA), and *Citrus* × *aurantium* L. (Fructus Aurantii, FA) (Chinese Pharmacopoeia Commission, 2020). Clinically, WFC is extensively utilized for preventing and managing precancerous lesions of gastric cancer (PLGC), chronic superficial gastritis, spleen-stomach weakness syndrome, and postoperative recovery in gastric malignancies (Chinese Pharmacopoeia Commission, 2020). In traditional Chinese medicine (TCM), PLGC results from spleen-stomach deficiency, phlegm-damp retention, and Qi stagnation with blood stasis, impairing digestion and potentially leading to cancer (Su et al., 2020; Pan et al., 2020). WFC addresses these pathologies by strengthening spleen Qi, regulating Qi flow, resolving turbidity, and eliminating pathogenic factors. Modern research confirms WFC’s efficacy, showing improvements in gastric mucosal atrophy, gut microbiota modulation, gastric histopathology enhancement, regulation of gastric acid and pepsin, and inflammation suppression via pathways like NF-κB (Bian et al., 2021; Xie et al., 2024). Nevertheless, detailed understanding of its pharmacodynamics and bioactive synergy in CAG—a critical precancerous stage—remains insufficient. Existing studies, primarily employing network pharmacology or single-target analyses (Bian et al., 2021; Wang et al., 2023a), fail to fully capture the comprehensive, multitargeted nature of traditional botanical medicine. Thus, rigorous, evidence-based ethnopharmacological studies are required to validate WFC’s therapeutic mechanisms and clinical potential in preventing gastric carcinogenesis.

The gut microbiota, comprising the collective genomes of gastrointestinal microorganisms, plays a crucial role in gastric disease pathogenesis, with beneficial and opportunistic microbes influencing gastric carcinogenesis (Kau et al., 2011; Yu et al., 2020; Nicholson et al., 2005; Fan and Pedersen, 2021; Ma et al., 2021). Untargeted metabolomics enables comprehensive profiling of endogenous metabolites, facilitating the discovery of differential metabolites and altered pathways in response to pathological stimuli (Kim et al., 2019; Asante et al., 2019). Concurrently, network pharmacology, integrating systems biology and computational analysis, has emerged as a pivotal tool for elucidating drug-disease interactions, particularly in Chinese medicine research (Fan et al., 2019). Liu et al. (2023) demonstrated that combining gut microbiota profiling, metabolomics, and network pharmacology effectively revealed the therapeutic mechanisms of Icariin in Alzheimer’s disease. Thus, integrated analysis of gut microbiota, metabolomics, and network pharmacology provides a comprehensive strategy for deciphering the mechanisms of therapeutic interventions.

In this study, network pharmacology was employed to predict the potential bioactive components of WFC. A CAG rat model was established using *N*-Methyl-N’-nitro-N-nitrosoguanidine (MNNG), followed by gut microbiota profiling and untargeted metabolomics to investigate the therapeutic mechanisms of WFC. Key targets were further validated through molecular docking and western blot analysis. This is the first study to elucidate the mechanistic basis of WFC in CAG treatment, providing new insights into its pharmacological actions and the gut microbiota–metabolite interactions underlying CAG progression and therapy.

## 2 Materials and methods

### 2.1 Material and reagents

High performance liquid chromatography (HPLC) grade methanol and acetonitrile were purchased from Tedia (Fairfield, OH, United States). Purified water was purchased from Wahaha (Zhejiang, Hangzhou, China). HPLC grade formic acid was procured from Fisher (Fair Lawn, NJ, United States). The WFC was purchased from Hangzhou Huqingyutang Pharmaceutical Co., Ltd, Zhejiang, China (Batch number: 2111230). Ranitidine Hydrochloride Capsules was purchased from Suzhou Homesun Pharmaceutical Co., Ltd, Zhejiang, China (Batch number: 306230127). Vitacoenzyme Tablets were procured from Guangxi Sunshine Pharmaceutical Co., Ltd, located in Guangxi, China, with batch number 230401. MNNG was obtained from Shanghai Macklin Biochemical Technology Co., Ltd, situated in Shanghai, China, with batch number C14631487.

### 2.2 Qualitative analysis of WFC

#### 2.2.1 Preparation of WFC extraction solution

Weifuchun Capsules (0.5 g) was ultrasonically treated with 10 mL of methanol-water (50:50, V/V). The solution was obtained after ultrasonic extraction for 40 min, centrifuged in a 4°C centrifuge at 15,000 rpm for 10 min, followed by filtration using a 0.22 μm filter membrane.

#### 2.2.2 Instrumental method

LC-30A ultra-high performance liquid chromatography (Shimadzu, Japan) and Triple TOF 5600^+^ mass spectrometer (AB Sciex, United States) were used to analyze the components of Weifuchun.

Chromatographic separation was performed on an Agilent Poroshell 120 SB-C18 column (2.1 × 100 mm, 2.7 Micron) at 40°C. The mobile phase consisted of 0.1% formic acid (A) and acetonitrile (B), and the flow rate was set at 0.3 mL/min and the injection volume was set at 5 μL. The elution conditions were as follows: 0–1 min, 5% B, 1–4 min, 5%–18% B; 4–16 min, 18%–46% B; 16–18 min, 46%–95% B; 18–20 min, 95% B; 20–21 min, 95%–5% B with pre-equilibrium of 5 min before injecting.

Mass spectrometry (MS) detection was done using electrospray ionization (ESI) in negative ion mode and Analyst TF 1.7 software with Information Dependent Acquisition (IDA) function and dynamic background subtraction. The turbo spray temperature was 550°C. Ion spray voltage floating (ISVF), declustering potential (DP), collision energy (CE) and collision energy spread (CES) were set at 5.5 kV, −80 V, −35, 15 eV. The ion source gas 1 (Gas1), ion source gas 2 (Gas2) and curtain gas (CUR) were 55 psi, 55 psi, 35 psi. The mass spectra in both primary and secondary ionization modes were examined over the mass range of 100–1400 Da and 50–1400 Da, respectively.

#### 2.2.3 Identification of compounds

China National Knowledge Infrastructure (CNKI) and PubChem databases were utilized to compile the chemical constituents of RG, IA, FA, and WFC in order to establish a comprehensive local database for WFC, encompassing names, precise molecular weights, and molecular formulas. Compounds were identified using the XIC Manager and IDA Explore functions within the PeakView 2.1 software platform developed by AB SCIEX in Concord, Ontario, Canada. Compounds with secondary mass spectrum discrepancies were chosen for structural identification. In the negative ion mode, a total of 99 compounds were discovered (Supplementary Table 1).

### 2.3 Network pharmacology analysis

The components, based on the previously identified and literature screened by SwissADME^1^, could be the active components of WFC, followed using the TCMSP^2^ database as a supplement. Compound structures were imported into the SwissTargetPredation^3^ to find WFC targets with probability > 0.1. CAG related targets were collected using OMIM^4^, PharmGKB^5^, Genecards^6^, and DisGenet^7^ databases.

The intersection targets were obtained by intersecting the WFC targets with the CAG related targets. Compound-target-disease interaction network was built by utilizing the WFC components and disease information associated with intersection targets in Cytoscape. The active ingredients were identified according to the degree value.

Online software String^8^ was utilized for the analysis of intersection targets, and the results were visualized using Cytoscape. Then, the hub gene was screened using Cytobubba.

### 2.4 Animal experiment and sample collection

Wistar male rats (4–5 weeks old) were obtained from Liaoning Changsheng Biotechnology Co., Ltd (License number: SCXK2020-0001). The animals were maintained in accordance with the guidelines established by the Medical Ethics Committee of Hebei Medical University, and the experimental protocol was approved by the committee (IACUC-Hebmu-2022134, 28 March 2022). The animals were housed under controlled conditions, including a 12 h light-dark cycle at a temperature of 22 ± 1.5°C and humidity of 50 ± 10%, with *ad libitum* access to food and water.

**FIGURE 1:**
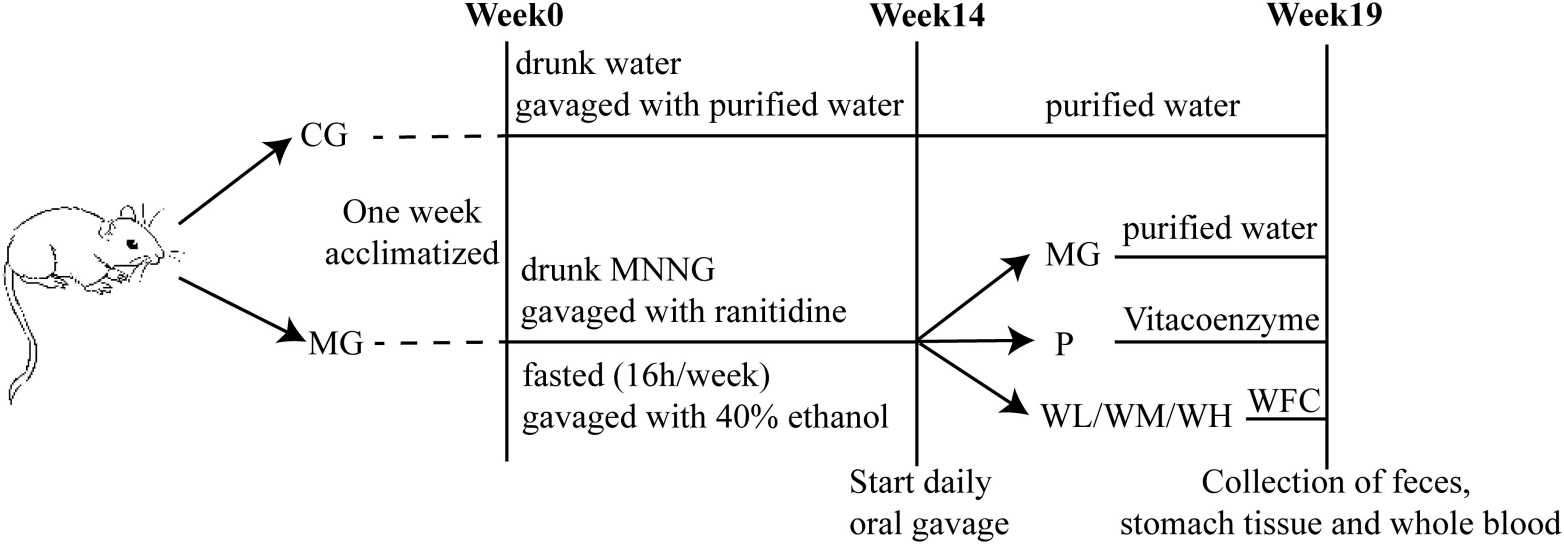
Schedule of animal treatment.

After a period of 7 days of adaptive feeding, a total of 40 rats were randomly divided into two groups: the control group (CG) and the model group (MG). The rats in the MG group drunk 120 μg/ml MNNG solution freely and administered ranitidine (3 mg/ml, 1 ml/100 g) via gavage, fasted once a week for 16 h, administered 40% (v/v) ethanol (1 ml/100 g) via gavage on the second day of fasting. From the 9 weeks, the MNNG concentration changed to 160 μg/ml for 6 weeks. The CAG model period lasted for 14 weeks. The rats in the CG group maintained free access to food and water and received an equivalent volume of purified water. Two rats in MG group were randomly selected for H&E staining at weeks 12 and 14 of modeling to observe whether the model was successfully established. After 14 weeks, 30 rats in MG group were randomly divided into five groups with six rats in each group: model group (MG), Vitacoenzyme group (P, 0.3 g/kg), WFC low-dose group (WL, 0.38 g/kg), WFC middle-dose group (WM, 0.76 g/kg), WFC high-dose group (WH, 1.52 g/kg). The dose administered to the WL group was determined by converting the clinical dose of WFC. The rats of the CG group and MG group administered purified water via gavage, and the P and WFC groups were given corresponding substances for 5 weeks (Figure 1).

The behavior and status of the rats were observed and the changes were recorded the changes. The rats’ daily weight was recorded throughout the experiment. The rats fasted for 24 h before the end of treatment. Rat fecal samples were obtained under sterile conditions at the end of the fast, flash-frozen with liquid nitrogen, and subsequently stored at −80°C for analysis of the gut microbiome. After collection of rat feces, all rats were anesthetized pentobarbital administered intraperitoneally. The whole blood was subsequently collected from the abdominal aorta for untargeted metabolomics analysis. Gastric tissue was collected after the blood collection for histopathological observation.

### 2.5 Histopathological observation

The gastric tissues underwent a series of preparatory steps including washing with saline, fixation with 4% paraformaldehyde, dehydration with gradient ethanol, cleaning with xylene, embedding in paraffin, sectioning at a thickness of 5.0 μm, and staining with hematoxylin and eosin (H&E). Subsequently, the pathological alterations in the gastric tissue were examined using a light microscope.

### 2.6 Gut microbiota analysis

Total genomic DNA of gut microbiota was extracted from rat feces, then libraries were created and 16S rRNA sequencing was performed. Illumina Novaseq 6,000 plateform was used to perform 16S rRNA sequencing. Trimmomatic v0.33 was used to filter the raw reads sequenced, and cutadapt 1.9.1 was used to identify and remove the primer sequences to obtain clean reads. Subsequently, the dada2 method in QIIME2 2020.6 was employed for denoising and obtaining non-chimeric reads. Consider reads obtained based on 97% sequence similarity as the operational taxonomic units (OTUs). QIIME software was used to calculate alpha diversity and beta diversity. Metastats analysis was used to study disparities in the abundance of microbial communities at the phylum and genus levels.

### 2.7 Untargeted microbiomics analysis

Whole blood was centrifuged at 3,500 rpm for 10 min at 4°C to collect the serum after 4 h of clotting. 55 ml serum was combined with 150 ml acetonitrile to induce precipitation. After vortexing for 5 min, the mixture centrifuged at 12,000 rpm at 4°C for 10 min. The supernatant was collected and evaporated using a vacuum centrifuge concentrator until dry. The dried residue was reconstituted with 100 μl of acetonitrile (1:1, V/V), vortexed for 1 min and centrifuged at 12,000 rpm for 10 min at 4°C, and the supernatant was collected for untargeted metabolomics analysis.

The system used for untargeted metabolomics analysis was the same as that used for qualitative analysis of WFC.

Chromatographic separation was performed on a Polar C18 (2.1 × 100 mm, 1.6 μm, Santa Clara, CA, United States) at 40°C. The mobile phase consisted of 0.1% formic acid (A) and acetonitrile (B), and the flow rate was set at 0.3 mL/min and the injection volume was set at 5 μL. The elution conditions were as follows: 0–1 min, 5% B; 1–4 min, 5%–45% B; 4–25 min, 45%–95% B; 25–26 min, 95%B; 26–27 min, 95–5% B with pre-equilibrium of 5 min before injecting.

The detection of MS was conducted utilizing an ESI source operating in positive and negative ion modes. The temperature of source was 550°C. The ISVF, CES, DP and CE of the positive ion mode were 5.5 kV, 15 eV, 60 V, and 35 eV, those of negative ion mode were −4.5 kV, 15 eV, −60 V, and −35 eV. Gas1, Gas2 and CUR were set at 55 psi, 55 psi and 35 psi. Primary and secondary mass spectra were scanned in the range of 100–1,250 Da and 50–1,250 Da, respectively.

In this research, a UHPLC-Q-TOF-MS/MS approach was utilized in conjunction with multivariate statistical analysis to analyze the metabolomics data. Progenesis QI (Umetrics, Sweden) was used to analyze raw metabolic data, including deconvolution, alignment, peak picking, and annotation of the peak. Independent Student’s *t*-test and fold change (FC) analysis were performed by importing the processed data into Metaboanalyst 5.0.^9^ Meanwhile, the processed data was upload into SIMCA-P 14.0 software (Umetrics, Uppsala, Sweden) for multivariate statistical analysis, including principal component analysis (PCA) and orthogonal partial least squares discriminant analysis (OPLS-DA). PCA is employed to analyze the clustering patterns among distinct datasets, while OPLS-DA may identify the variables that exhibit differences between groups and also calculate their VIP values. The quality of the OPLS-DA model was assessed through examination of coefficient of determination (R^2^, Q^2^) and 200 permutation tests. The differential metabolites met the conditions of VIP > 1, *p* < 0.05 and FC ≥ 1.2 or ≤ 0.8. Then, the differential metabolites were identified using the Human Metabolome Database (HMDB) and Kyoto Encyclopedia of Genes and Genomes (KEGG). Lastly, the metabolic pathways of differential metabolites were analyzed via the MetaboAnalyst 5.0.

### 2.8 Network construction and molecular docking analysis

The network was constructed based on untargeted metabolomics data using Metscape. The KEGG IDs of differential metabolites were imported into Metscape to construct an interaction network of metabolite-response-enzyme-gene to demonstrate the relationships among metabolites, reactions, enzymes, genes and obtain metabolome-related genes.

The primary focus of this study was analyzed using a combination of network pharmacology and untargeted metabolomics. The interaction between the active components and main targets was assessed using AutoDock Vina 1.2.2. Compound structures were obtained from PubChem, while the crystal structures of key targets were sourced from the Protein Data Bank (PDB^10^).

### 2.9 Integrated analysis of gut microbiota and untargeted metabolomics

Through the use of the Spearman correlation analysis method, the connection between significant differential flora and significant differential metabolites was visually represented through a correlation coefficient matrix heat map.

### 2.10 Statistical analysis

The data was presented as mean ± standard deviation (SD), and statistical comparisons between the two groups were conducted using a *t*-test. The Origin 2021 and BMKCloud Platform were used for graphics. Statistical significance was operationally defined as significance levels of *p < 0.05, **p < 0.01, and ***p < 0.001.

### 2.11 Detection of IL6, AKT1, and PLA2G1B protein expression by western blot

Gastric tissue samples (20 mg) were homogenized in lysis buffer to extract proteins. The protein concentration was determined using the BCA assay. Following denaturation in a boiling water bath for 10 min, 10 μL of the protein sample was loaded onto a gel (160 V, 40 min) for electrophoresis, after which the proteins were transferred to a polyvinylidene fluoride (PVDF) membrane. After transfer, the membrane was blocked using a rapid blocking solution and incubated overnight at 4°C with primary antibodies against IL6, AKT1, PLA2G1B, and GAPDH (dilution ratios of 1:4000, 1:10000, 1:2000, and 1:50000, respectively). The membrane was then incubated with the corresponding secondary antibody (dilution ratio of 1:5000) at 37°C for 1 h. Protein bands were visualized using ECL chemiluminescent substrate and imaged with a chemical imaging system. GAPDH was used as the internal control, and the band intensities were analyzed using ImageJ software.

## 3 Results

### 3.1 Network pharmacology analysis

**FIGURE 2:**
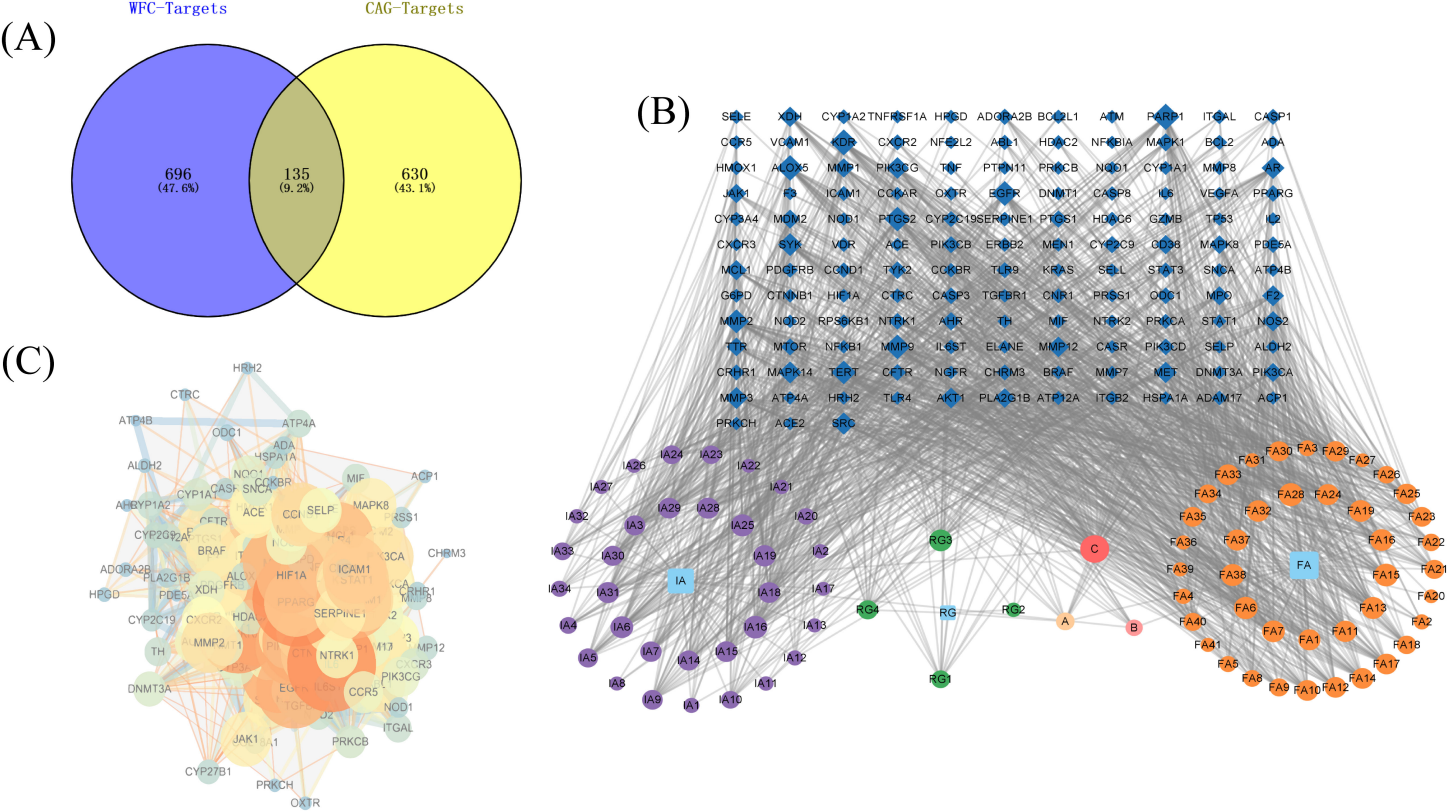
Network pharmacology analysis of Weifuchun Capsule (WFC) treatment for chronic atrophic gastritis (CAG). **(A)** Venn diagram of WFC and CAG intersection targets. **(B)** Component-disease-target network. **(C)** PPI network.

A total of 107 compounds were found based on experimental data, literature and databases, which can be potentially active compounds for the treatment of CAG with WFC. 831 potential targets were predicted for these active compounds, and 135 intersection targets were obtained after taking intersections with 765 disease targets (Figure 2A). Detailed information on the compositions containing intersecting targets is in Supplementary Table 2. Subsequently, based on the intersection targets a component-disease-target network was established (Figure 2B), then based on the degree value potential active ingredients were screened, and the top-ranked ingredients were quercetin, narcotine, glaucocalyxinD, deacetylnomilin, 5,7,4-trimethoxyflavone, methylrosmarinate, byakangelicol, tangeretin, protopanaxatriol and cirsiliol. The PPI network of intersection targets visualized in Cytoscape (Figure 2C). Then, Cytohubba was employed to analyze the hub genes, which included IL6, TNF, AKT1, TP53, NFKB1, EGFR, STAT3, SRC, BCL2 and CASP3.

### 3.2 Pharmacodynamic study of WFC against CAG rats

Rats in CG group had normal food intake and well mental status. Rats in MG group have decreased food intake, poor mental status, lethargy and significantly reduced activity. After treatment with Vitacoenzyme or WFC, all of these symptoms improved.

**FIGURE 3:**
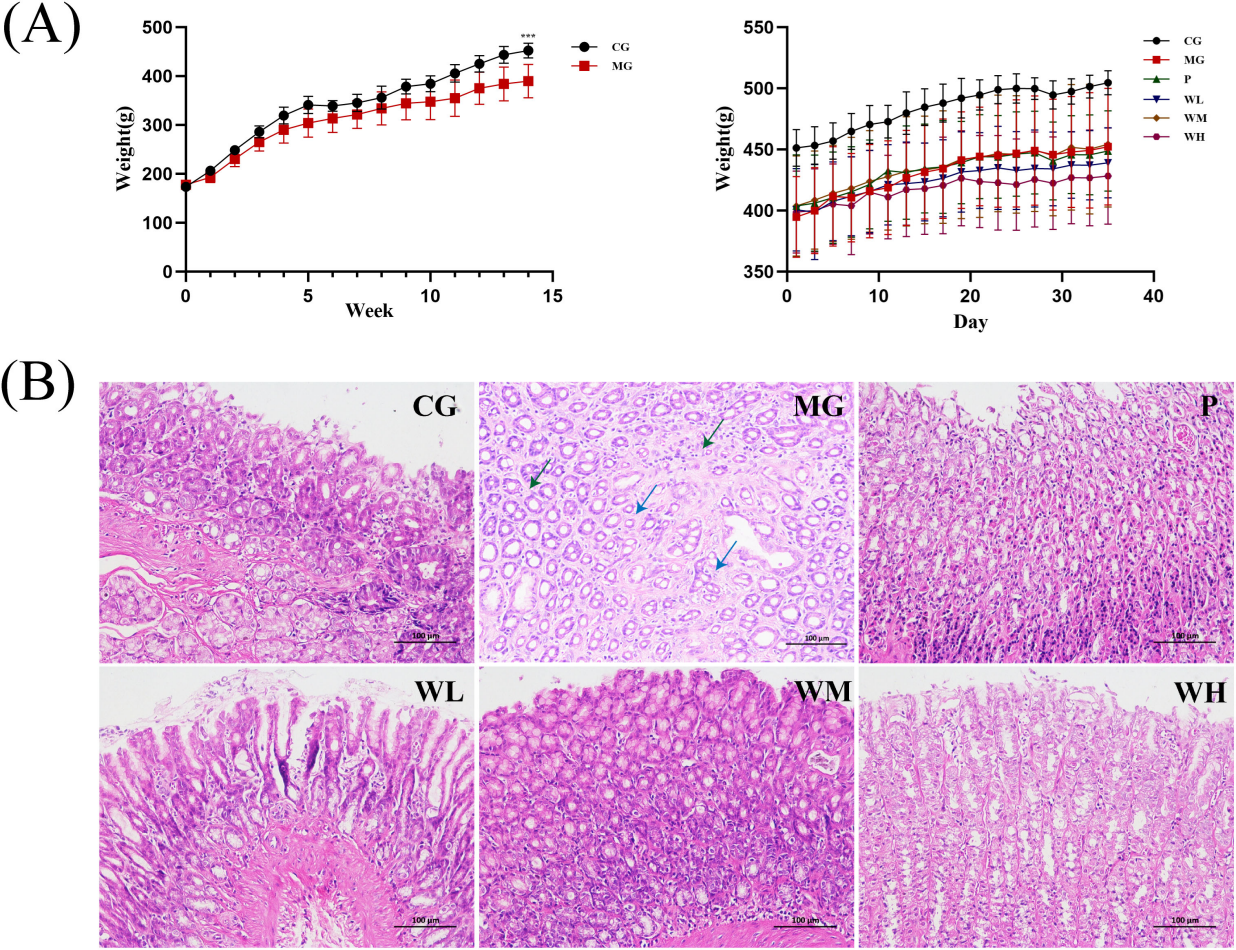
Pharmacodynamic evaluation of Weifuchun Capsule (WFC) treatment for chronic atrophic gastritis (CAG). **(A)** Body weight fluctuations in rats during the experimental period. **(B)** Gastric histopathological sections in all the experimental groups (***P < 0.001).

Loss of weight is a common CAG symptom. Prior to modeling, no statistically significant differences were found between the CG group and the MG group. After modeling, it was discovered that the mean weight of the MG group was substantially lower than the CG group. After treatment with Vitacoenzyme or WFC, weight recovery was most pronounced in the WM group (Figure 3A).

The findings of H&E staining showed that, compared with the CG group, the MG group exhibited edema in the lamina propria, atrophy of the gastric glands, volume reduction, irregular shape, sparse arrangement (blue arrowheads) and a small amount of lymphocytic infiltration in the interstitium (green arrowheads). After treatment with Vitacoenzyme or WFC, the degree of gastric mucosal damage was improved, with the best treatment effect in the WM group (Figure 3B).

### 3.3 Gut microbiota analysis

Fecal samples were analyzed from rats in the CG, MG and WM group by Illumina NovaSeq platform, in order to determine the community structure and species composition following WFC treatment.

**FIGURE 4:**
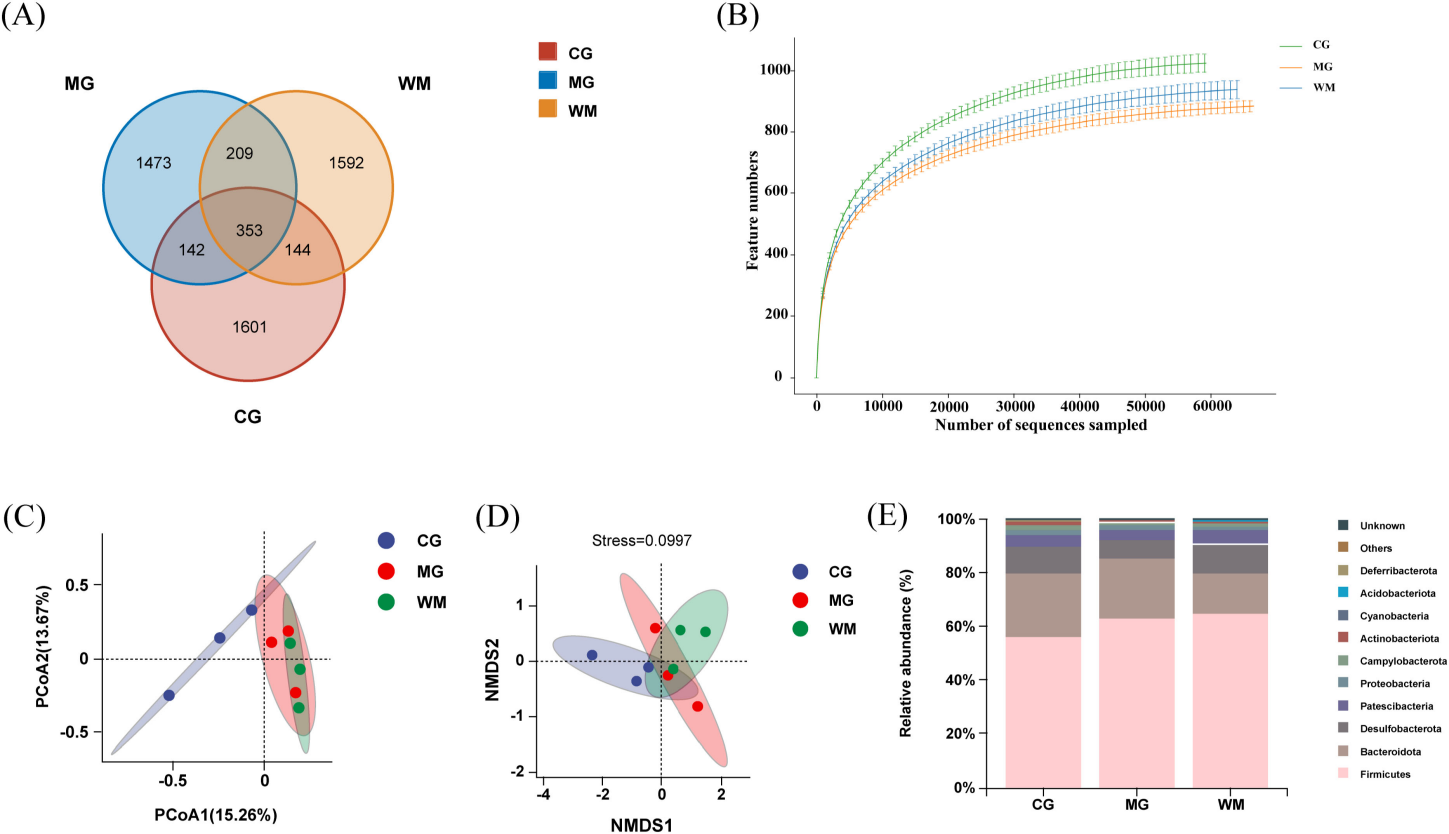
Gut microbiota analysis of Weifuchun Capsule (WFC) treatment for chronic atrophic gastritis (CAG). **(A)** Venn diagram of three groups based on operational taxonomic units (OTUs); **(B)** Rarefaction curve; **(C)** Principal coordinates analysis (PCoA) analysis score plot; **(D)** Non-metricmulti-dimensional scaling (NMDS) analysis score plot; **(E)** Histogram of relative abundance at the phylum level.

The Venn diagram illustrated that a total of 353 OTUs overlapped among the three groups, 1,601 distinct OTUs in the CG group, 1,473 distinct OTUs in the MG group, 1,592 distinct OTUs in the WM group (Figure 4A). Rarefaction curves exhibit a reasonably low slope, proving that the sample size is enough (Figure 4B).

Alpha diversity analysis based on Chao1, ACE, Shannon, and Simpson indices were utilized to study the differences in intestinal flora among the CG, MG and WM groups (Table 1). Compared with the CG group, Chao1, ACE, Shannon and Simpson indices of MG group were decreased, indicating that diversity and richness were altered in CAG rats. However, the differences observed in these indices between groups were not statistically significant. The values of all four indices were elevated above normal levels after WFC treatment, indicating that WFC played a protective role against microbial diversity and richness in CAG rats, although this effect did not reach statistical significance.

**TABLE 1:** The alpha diversity indices for three groups of rats fecal samples (mean ± SD).

Group	Chao1	ACE	Shannon	Simpson
CG	914.10 ± 198.35	916.71 ± 198.96	7.6163 ± 0.2668	0.9863 ± 0.0017
MG	889.89 ± 32.41	894.96 ± 32.66	7.5591 ± 0.0971	0.9853 ± 0.0053
WM	944.80 ± 55.69	949.82 ± 57.26	7.5549 ± 0.2063	0.9831 ± 0.0068

Beta diversity analysis based on principal coordinates analysis (PCoA) and non-metricmulti-dimensional scaling (NMDS) was employed to address the variations in the microbiota composition amongst groups. Figure 4C shows the significant shifts between CG and MG groups, indicating that MNNG affected the microbiota composition of rats to some extent. After WFC treatment, compared with MG group, the WM group did not exhibit distinct separation, indicating that WFC partially improved the gut microbiota of rats with CAG. In addition, NMDS also confirmed these results (Figure 4D).

In order to conduct a more thorough examination of the influence of WFC on the makeup of gut microbiota, alterations in the relative abundance of gut microbiota were assessed at the phylum level (Figure 4E). Investigations indicated that *Firmicutes*, *Bacteroideyes*, and *Desulfobacterota* were the three most prevalent phyla among all three groups.

**FIGURE 5:**
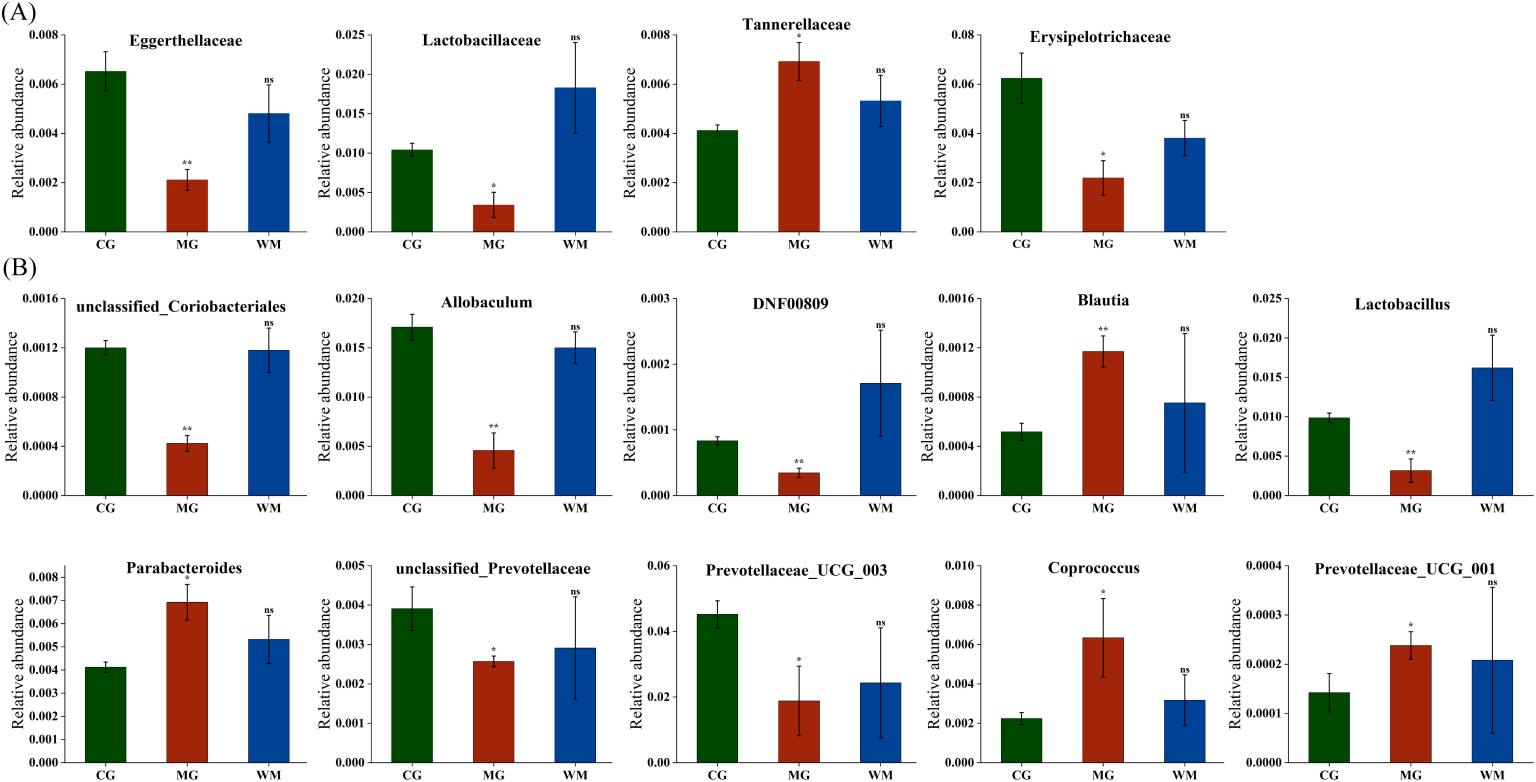
Effect of chronic atrophic gastritis (CAG) on the relative abundance of gut microbiota at family **(A)** and genus **(B)** level. Data were presented as mean ± SD (*n* = 3). *p < 0.05; **p < 0.01.

Metastats analysis was used to search for the main taxa of statistical significance at the family and genus levels (Figure 5). A total of four distinct families and 10 distinct genera were discovered between the CG and MG groups (*p* < 0.05), and there were no statistical differences between CG and WM groups. At the familial level, compared to the CG group the relative abundance of *Eggerthellaceae*, *Lactobacillaceae*, and *Erysipelotrichaceae* was found to be decreased in the MG group, whereas *Tannerellaceae* exhibited an increase. At the genus level, compared with the CG group, the relative abundance of *unclassified_Coriobacteriales*, *Allobaculum*, *DNF00809, Lactobacill, usunclassified_Prevotellaceae*, and *Prevotellaceae_UCG_003* were decreased, while *Blautia*, *Parabacteroides*, *Coprococcus*, and *Prevotellaceae_UCG_001* were increase in the MG group. After WFC treatment, the relative abundance of all flora was adjusted to normal levels. These results demonstrated that MNNG significantly altered certain flora and adjusted the dysbiotic mycobiome to normal levels after WFC treatment.

### 3.4 Untargeted metabolomics analysis

**FIGURE 6:**
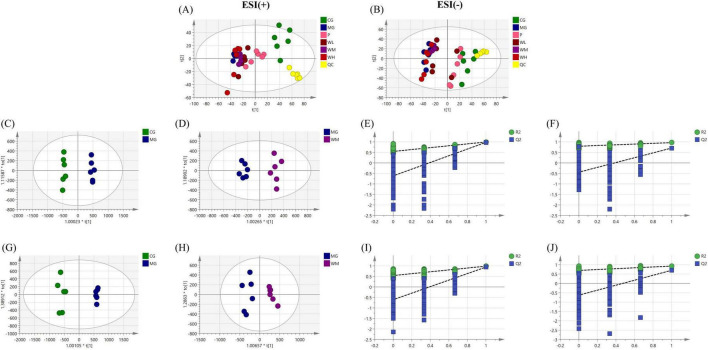
Untargeted metabolomic analysis of Weifuchun Capsule (WFC) treatment for chronic atrophic gastritis (CAG). **(A,B)** Principal component analysis (PCA) score plots in positive **(A)** and negative **(B)** ion modes. **(C,G)** The orthogonal partial least squares discriminant analysis (OPLS-DA) score plot in positive **(C)** and negative **(G)** ion modes(CG vs. MG). **(D,H)** The OPLS-DA score plot in positive **(D)** and negative **(H)** ion modes (MG vs. WM). **(E,I)** Permutation test of the OPLS-DA model in positive **(E)** and negative **(I)** ion modes (CG vs. MG). **(F,J)** Permutation test of the OPLS-DA model in positive **(F)** and negative **(G)** ion modes (MG vs. WM).

The PCA score plots showed that there was a clear tendency of separation between the CG and MG groups in both positive and negative ion modes (Figures 6A, B). After WFC treatment, the administered groups showed a tendency to cluster closer to the CG group while separating from the MG group, indicating that WFC is therapeutically effective. OPLS-DA identified distinguishing metabolites affecting classification (Figures 6C, D, G, H). Within the OPLS-DA model, R^2^X, R^2^Y, and Q^2^ are significant predictive parameters. The model is more stable and reliable the closer R^2^Y and Q^2^ are near 1. In general, the model can be considered valid when R^2^Y > 0.5 and Q^2^ > 0.5. R^2^Y and Q^2^ of the OPLS-DA model were 0.994 and 0.977 in CG vs. MG, and 0.965 and 0.705 in MG vs. WM in positive ion mode, while were 0.988 and 0.944 in CG vs. MG, and 0.928 and 0.702 in MG vs. WM in negative ion mode (Table 2). The aforementioned results indicate a considerable distinction between the MG group and the CG and WM groups, highlighting a notable disparity in the metabolic processes of the MG group compared to the other groups. And, the 200 times permutation test showed that is not overfit (Figures 6E, F, I, J).

**TABLE 2:** Predictive parameters in positive and negative ion modes.

	Positive ion mode		Negative ion mode	
	CG vs. MG	MG vs. CG	CG vs. MG	MG vs. CG
R^2^Y	0.994	0.965	0.988	0.944
Q^2^	0.977	0.705	0.928	0.702

A total of 89 differential metabolites were screened between the CG and MG groups, compared to the CG group, 44 and 45 of 89 differential metabolites showed an apparent increase and decrease. 53 differential metabolites were screened between the MG and WM groups, there were seven metabolites upregulated and 46 metabolites downregulated in the WM group compared to the MG group. WFC significantly modulated 21 differential metabolites and reversed 20 metabolites of them, demonstrating the effectiveness of the treatment (Table 3).

**TABLE 3:** Differential metabolites between control group (CG) and model group (MG) rats and between MG and WM rats.

No.	Ion mode	Identification	Formula	CG vs. MG		Trend	*P*	MG vs. WM		Trend	*P*
				VIP	FC			VIP	FC		
1	+	Indole-3-methyl acetate	C_11_H_11_NO_2_	1.83965	4.0709	↓	***	1.63948	0.59591	↑	***
2	+	5-Hexyl-3-methyl-2-furannonanoic acid	C_20_H_34_O_3_	1.17537	2.1892	↓	*	1.40487	2.0081	↓	**
3	+	Vaccenyl carnitine	C_25_H_47_NO_4_	1.11974	0.73046	↑	*	1.62434	1.5867	↑	**
4	+	PGP(18:1(9Z)/18:1(9Z))	C_42_H_80_O_13_P_2_	1.68979	0.29759	↑	**	1.40851	1.458	↓	*
5	+	Stearoylcarnitine	C_25_H_50_NO_4_	1.10751	0.26682	↑	**	1.16834	1.5752	↓	**
6	+	PA(15:0/22:1(13Z))	C_40_H_77_O_8_P	9.26945	0.055057	↑	**	9.59915	2.0154	↑	*
7	+	PC(16:0/20:5(5Z,8Z,11Z,14Z,17Z))	C_44_H_78_NO_8_P	8.28055	0.089034	↑	***	8.77378	1.9781	↑	*
8	+	PE(15:0/22:2(13Z,16Z))	C_42_H_80_NO_8_P	13.5278	0.084346	↑	***	13.9669	1.881	↑	*
9	+	PC(16:1(9Z)/18:1(11Z))	C_42_H_80_NO_8_P	13.0845	0.079149	↑	***	12.8065	1.7969	↑	*
10	+	PE-NMe(18:0/18:2(9Z,12Z))	C_42_H_80_NO_8_P	10.7121	0.056403	↑	***	10.6551	1.8282	↑	*
11	+	PC(18:2(9Z,12Z)/16:0)	C_42_H_80_NO_8_P	8.71901	0.18993	↑	**	9.09954	1.7359	↑	*
12	+	PC(20:5(5Z,8Z,11Z,14Z,17Z)/16:0)	C_44_H_78_NO_8_P	3.23503	0.25343	↑	**	3.6806	1.8211	↑	**
13	+	PE(20:1(11Z)/18:0)	C_43_H_84_NO_8_P	3.47789	0.11323	↑	**	2.29252	1.4212	↑	*
14	+	PE-NMe2(18:2(9Z,12Z)/22:5(4Z,7Z,10Z,13Z,16Z))	C_47_H_80_NO_8_P	1.7839	0.02288	↑	***	1.13673	1.4804	↓	*
15	+	PE-NMe2(18:3(6Z,9Z,12Z)/22:6(4Z,7Z,10Z,13Z,16Z,19Z))	C_47_H_76_NO_8_P	1.38336	0.37701	↑	*	1.97633	1.9747	↑	*
16	+	PA(20:0/18:1(9Z))	C_47_H_76_NO_8_P	1.4573	0.053302	↑	***	1.34572	1.6881	↓	*
17	+	Ganglioside GM3 (d18:1/20:0)	C_61_H_112_N_2_O_21_	1.16707	3.6412	↓	***	1.13366	0.57175	↑	**
18	−	Stearoylglycine	C_20_H_39_NO_3_	1.43442	0.11606	↓	**	1.25823	1.6631	↑	**
19	−	FAHFA(18:1(9Z)/9-O-18:0)	C_36_H_68_O_4_	1.24633	0.13528	↓	**	2.14803	3.4454	↑	**
20	−	N-Stearoyl GABA	C_22_H_43_NO_3_	1.41511	0.31845	↑	**	1.27598	1.4534	↓	**
21	−	(±)-Tryptophan	C_11_H_12_N_2_O_2_	1.93037	0.35772	↑	***	1.17915	1.249	↓	*

**FIGURE 7:**
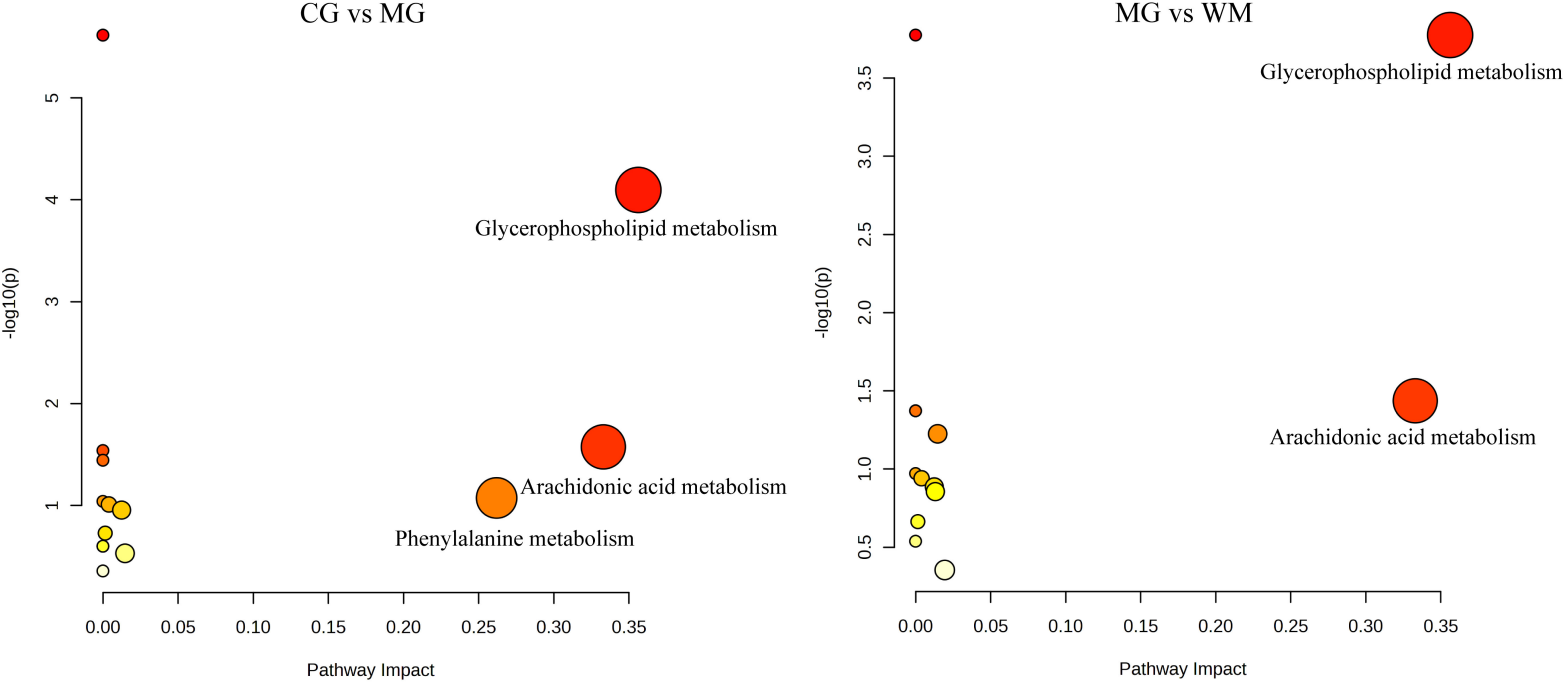
Pathway analysis of metabolites with MetaboAnalyst 5.0.

A total of 89 and 53 differential metabolites were mapped to the KEGG database to investigate the pathways of MNNG-induced CAG and WFC treatment, respectively (Figure 7). Three primary metabolic pathways were altered in the MG group, including glycerophospholipid metabolism, arachidonic acid metabolism, and phenylalanine metabolism. Treatment with WFC notably influenced glycerophospholipid metabolism and arachidonic acid metabolic pathways.

### 3.5 Network analysis

**FIGURE 8:**
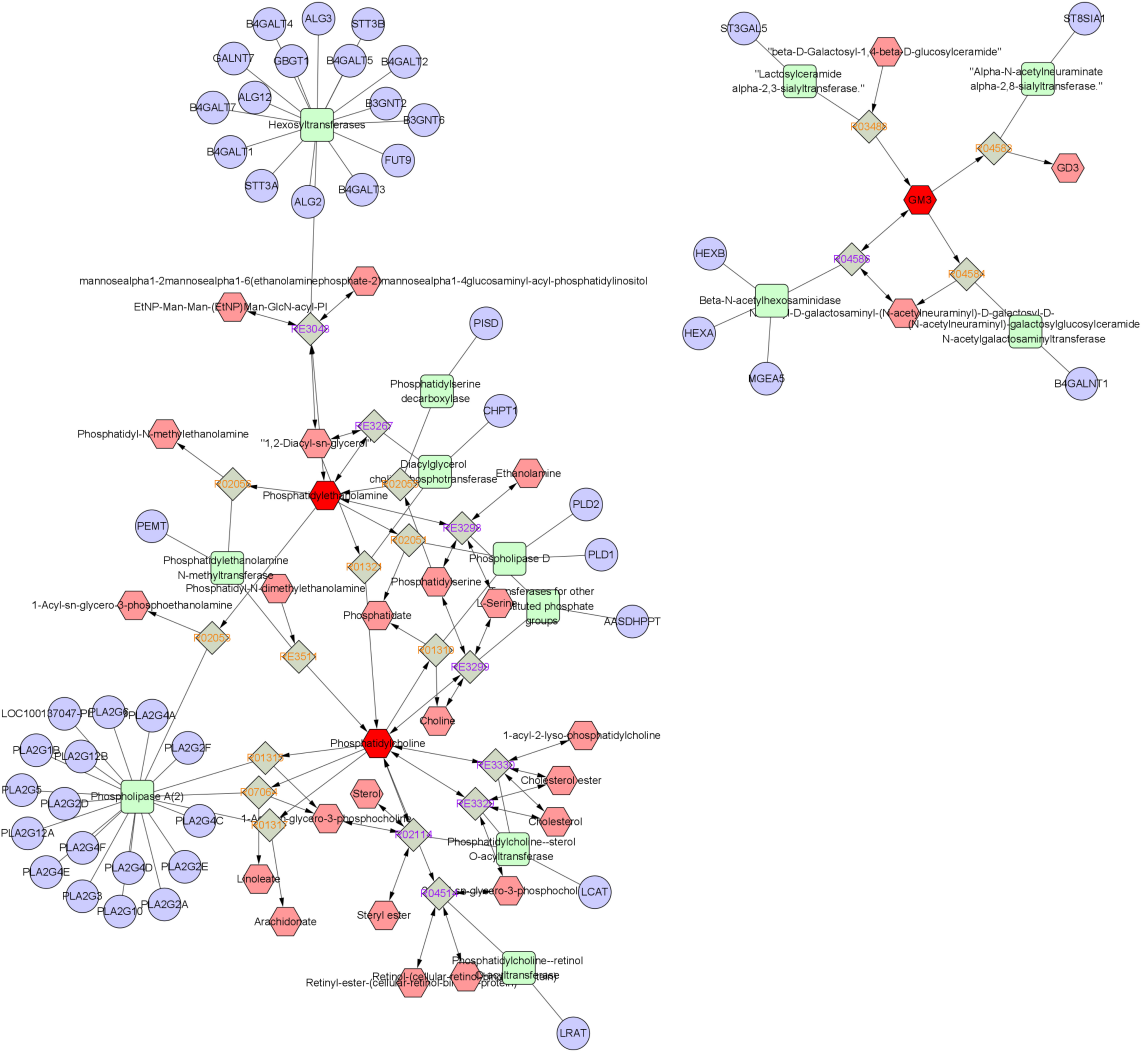
A metabolic-reaction-enzyme-gene network.

To further investigate the mechanism of WFC treating CAG, a metabolite-response-enzyme-gene network was constructed (Figure 8). KEGG IDs of 21 differential metabolites obtained from untargeted metabolomics were imported into Metscape to construct the network. There were 110 nodes containing 28 metabolites (including three input biomarkers), 22 reactions, 13 enzymes and 47 genes.

### 3.6 The effect of WFC on the expression of IL6, AKT1, and PLA2G1B proteins in rat gastric tissue

Combining the targets obtained from network pharmacology and network analysis, a main target was obtained: IL6, AKT1, and PLA2G1B. PLA2G1B (PDB ID: 3ELO), which can be a key target for WFC treating CAG.

**FIGURE 9:**
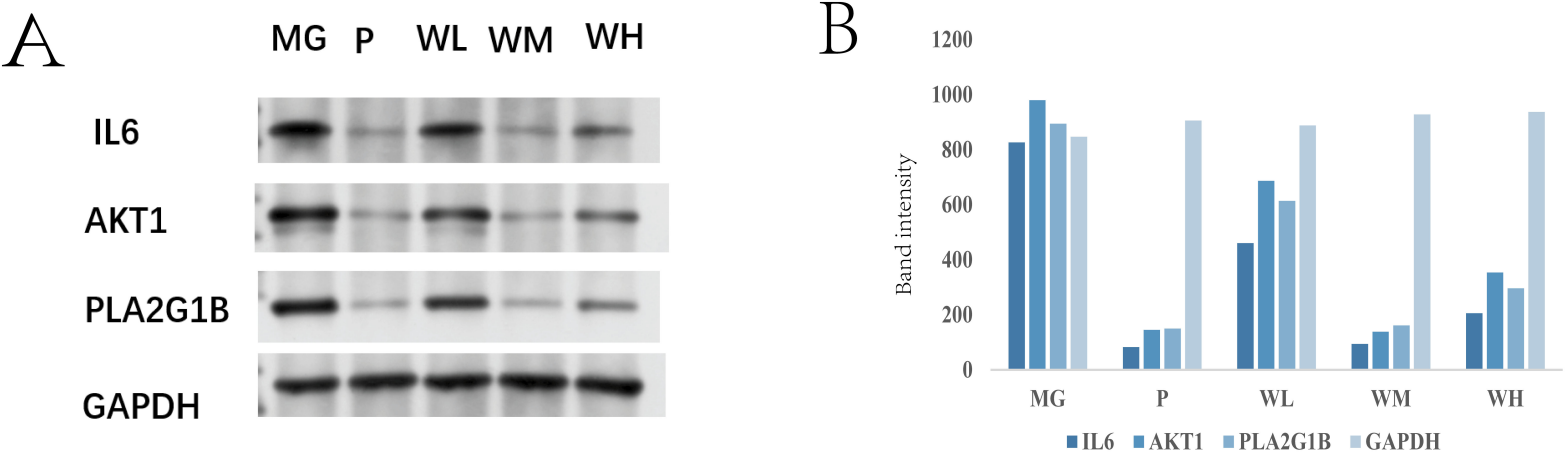
Expression of IL6, AKT1, and PLA2G1B proteins in gastric tissues of rats in each group ($\bar{\text{x}}$ ± *s*, *n* = 3) **(A,B)**.

Western blot analysis (Figure 9) revealed that, compared with the MG group, the expression levels of IL6, AKT1, and PLA2G1B proteins were significantly reduced in the gastric tissue of rats from the WFC group (WM, WH) (*P* < 0.005). Compared with the WL group, the expression of IL6, AKT1, and PLA2G1B proteins was significantly lower in the WM and WH groups (*P* < 0.05). In comparison with the P group, the protein expression in the WM group was similar to that in the P group (*P* < 0.005). When comparing PLA2G1B protein expression between the model groups, the WH group exhibited lower protein expression than the WL group, and the WM group showed lower expression than the WH group. These results indicate that WFC can significantly modulate the inflammatory factors IL6, AKT1, and PLA2G1B, with a particularly notable effect on the regulation of PLA2G1B protein expression. WFC may thus influence the inflammatory response in chronic atrophic gastritis.

### 3.7 Molecular docking analysis

**FIGURE 10:**
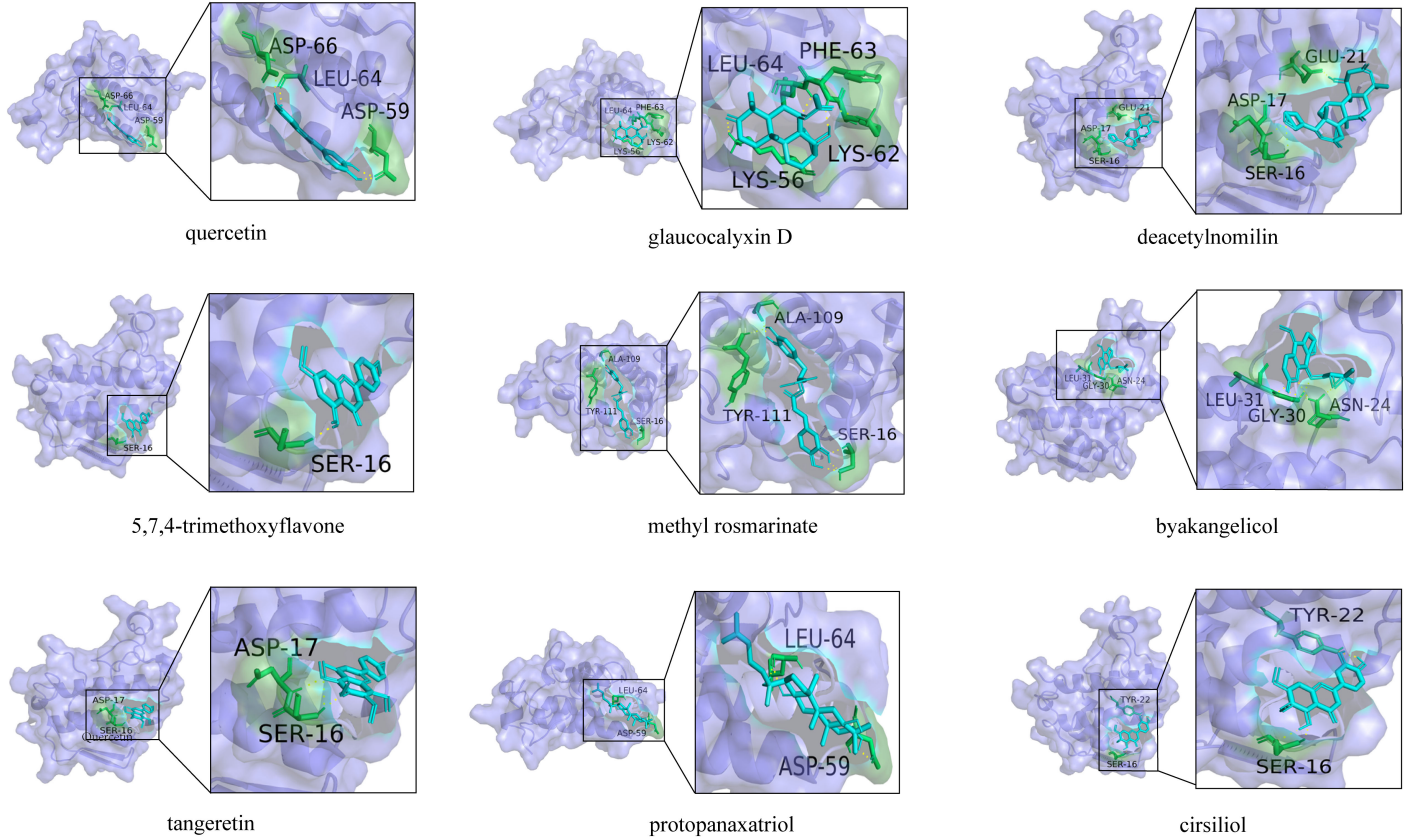
Docking mode between core proteins and associated key targets.

To further screen the active components of WFC, molecular docking was performed with proteins and active ingredients obtained from network pharmacology to predict their binding energies (Figure 10). Table 4 displays the findings of the docking analysis. Affinity less than −5 kcal/mol can be considered that the active ingredients bind intimately to the main target, and it is reasonable to believe that these substances are crucial to the WFC therapy of CAG.

**TABLE 4:** The docking scores of PLA2G1B and nine active ingredients.

No.	Ingredient	Binding energy (kcal mol^–1^)
1	Quercetin	−6.41
2	GlaucocalyxinD	−7.57
3	Deacetylnomilin	−7.14
4	5,7,4-trimethoxyflavone	−6.27
5	Methylrosmarinate	−6.18
6	Byakangelicol	−6.79
7	Tangeretin	−5.93
8	Protopanaxatriol	−9.05
9	Cirsiliol	−6.62

### 3.8 Correlation between gut microbiota and untargeted metabolomics

**FIGURE 11:**
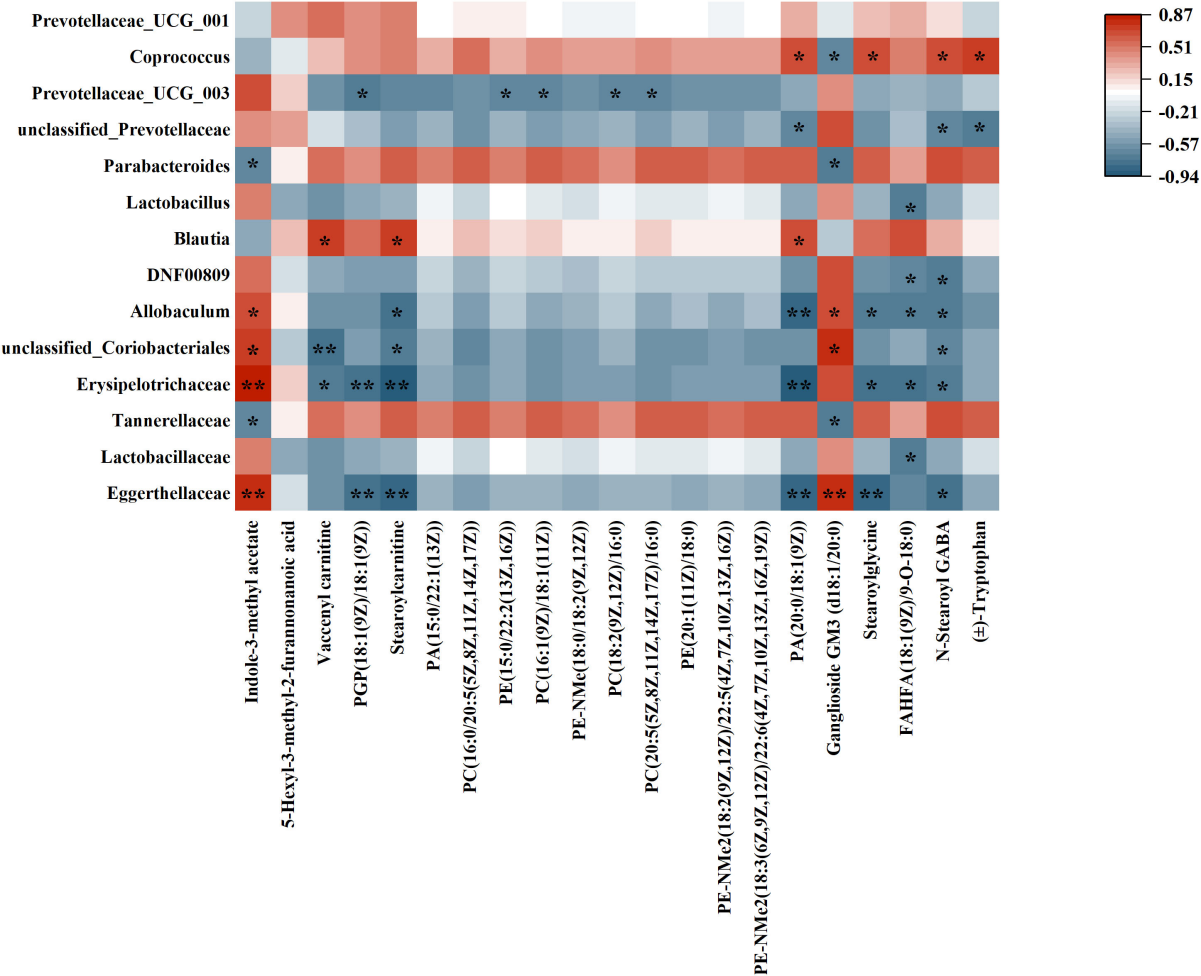
Heatmap of correlation between differential flora and differential metabolites. (*p < 0.05; **p < 0.01).

Spearman correlation analysis was utilized to investigate the relationship between differential flora and differential metabolites (Figure 11). The gut microbiota consisted of 14 distinct types of flora, whereas the untargeted metabolomics identified 21 distinct types of metabolites. Spearman correlation analysis was performed on these flora and metabolites, and the results were visualized in a heatmap. For example, *Eggerthellaceae* showed positive correlation with Indole-3-methyl acetate and Ganglioside GM3 (d18:1/20:0); showed negative correlation with PGP (18:1(9Z)/18:1(9Z)), Stearoylcarnitine, PA (20:0/18:1(9Z)), Stearoylglycine, and N-Stearoyl GABA. *Lactobacillaceae* showed negtative correlation with FAHFA(18:1(9Z)/9-O-18:0). *Tannerellaceae* showed negtative correlation with Ganglioside GM3 (d18:1/20:0). *Erysipelotrichaceae* showed positive correlation with Indole-3-methyl acetate; showed negative correlation with Vaccenyl carnitine, PGP(18:1(9Z)/18:1(9Z), Stearoylcarnitine, PA(20:0/18:1(9Z)), Stearoylglycine, FAHFA(18:1(9Z)/9-O-18:0) and N-Stearoyl GABA.

## 4 Discussion

In this study, the chemical compositions of WFC were identified by UPLC-Q-TOF-MS/MS and the active components of WFC for treating CAG were predicted using network pharmacology. Subsequently, MNNG-induced CAG rat model was established to to assess the therapeutic impact of WFC in treating CAG. The findings from untargeted metabolomics and gut microbiota indicated that WFC could reverse the imbalance of differential metabolites and differential flora in CAG rats, demonstrating the effectiveness of the treatment and exploring the molecular mechanisms, which were validated by molecular docking.

A total of 99 chemical constituents were identified in WFC using UPLC-MS/MS, then network pharmacological analysis was performed to search for active ingredients and hub genes. Quercetin exerts anticancer properties on cancer cells and tumors through the modulation of the PI3K/Akt/mTOR, Wnt/β-catenin and MAPK/ERK1/2 pathways (Reyes-Farias and Carrasco-Pozo, 2019). Glaucocalyxin D has strong Cytotoxic activity against human tumor cells (Liu et al., 2017). Protopanaxatriol exhibits notable anti-inflammatory properties, and its possible mechanism is to ameliorate inflammatory diseases through the suppression of pro-inflammatory cytokines IL-1β and TNF-α, as well as the inhibition of the interaction between LPS and TLR4 on macrophages (Lee et al., 2015). Moreover, protopanaxatriol achieves its anti-tumor effect by promoting the binding of the tumor gene P53 to DNA (Wang et al., 2020).

Research has demonstrated that the occurrence of CAG results in alterations in the composition of the gut microbiota (Huang et al., 2021; Zhou et al., 2022). In this study, altered diversity and abundance in CAG rats, returned to normal after WFC treatment. At the phylum level, there was some difference in the composition of intestinal flora among the groups of rats, suggesting that the intestinal flora of CAG rats was disturbed to a certain extent and its species structure was changed, but there was no statistical difference. *Firmicutes* and *Bacteroidetes* represent the predominant gut microbiota in both human and animal, and the dysregulation of their *Firmicutes*/*Bacteroidetes* ratios can be considered as a dysbiosis of the gut microbiota (Stojanov et al., 2020). Li et al. (2022b) discovered that an elevation in the *Firmicutes/Bacteroidetes* ratio altered the gut environment, thereby improving immune response and promoting gastrointestinal wellbeing. In this study, the WM group had a considerably larger *Firmicutes*/*Bacteroidetes* ratio than the CG and MG groups, which is consistent with the above experimental results.

Metastats was used to study differential flora at the phylum and genus levels. *Lachnospiraceae* synthesizes significant quantities of short-chain fatty acids (SCFA), which maintain the intactness of the gastric mucosa and inhibit the proliferation of harmful intestinal bacteria by inhibiting the release of inflammatory factors to maintain the health of the organism (Fung et al., 2017; Guo et al., 2022). In a study of indomethacin-induced gastric ulcers in rats, a decrease in the relative abundance of *Lachnospiraceae* was reversed after RD-6 treatment, aligning with the findings of the current research (Feng et al., 2023). Some studies suggested that *Eggerthellaceae* is related to CAG, but its mechanism in CAG is not clear (Huang et al., 2022; Wu et al., 2022). A study demonstrated a notable reduction in the relative abundance of *Eggerthellaceae* in Salmonella typhimurium-Challenged mice, which was reversed after cinnamaldehyde treatment, demonstrating that the relative abundance of *Eggerthellaceae* was linked with impaired gut barriers and the development of gastrointestinal disease (Wang et al., 2021b). *Tannerellaceae* are considered to be harmful bacteria that threaten the health of host, and there is no direct evidence of a relationship between *Tannerellaceae* and CAG, but a study has shown that the abundance of *Tannerellaceae* was significantly elevated in *Escherichia coli* infected mice, a finding that aligns with the outcomes of this study (Wang et al., 2023b). The relative abundance of *Erysipelotrichaceae* is associated with gastrointestinal disorders. Research has shown that in gut microbiota studies of cisplatin-induced intestinal injury in rats, the relative abundance of *Erysipelotrichaceae* was reduced, and reversed following treatment with *Poria cocos.* The possible mechanism is that *Erysipelotrichaceae* can produce butyrate, which can decrease endothelial permeability and improve intestinal function (Pozuelo et al., 2015; Zou et al., 2021).

The findings from the untargeted metabolomics suggested that glycerophospholipid metabolism and arachidonic acid metabolism were the major metabolic pathways involved in the treatment of CAG with WFC. Glycerophospholipids are widely distributed in nature and constitute the predominant class of phospholipids in the human body (Sheng et al., 2022). Glycerophospholipids have a variety of biological functions, and disruptions in their metabolic network can lead to numerous diseases, such as rheumatoid arthritis, depression, atherosclerosis, acute ulcerative colitis and so on Jian et al. (2023), Tian et al. (2022), Yang et al. (2024), Yuan et al. (2020). Metabolism was disrupted and the glycerophospholipid metabolic pathway was affected in CAG rats (Chen et al., 2020). Arachidonic acid is crucial and necessary fatty acid in human beings, with its metabolic pathway mainly including three pathways: cyclooxygenase (COX), lipoxygenase (LOX) and cytochrome P450 (CYP450) (Wang et al., 2021a). COX-2, an isoenzyme of COX, can encourage the production of prostaglandins from arachidonic acid, which ultimately produce a range of inflammatory mediators (Maier et al., 1990). Research has demonstrated a significant upregulation of COX-2 in individuals with gastric atrophy, intestinal metaplasia, and gastric cancer, indicating a potential role of COX-2 in the pathogenesis of gastric carcinogenesis (Sung et al., 2000). Furthermore, accumulating evidence has indicated that CAG rats exhibit dysregulation in arachidonic acid metabolism, resulting in the development of disorders (Tong et al., 2021; Xi et al., 2021).

Combining the intersecting targets of network pharmacology and the genes obtained from network analysis to find a main target: PLA2G1B. The results of untargeted metabolomics were validated using molecular docking to calculate affinity energies. Western blot analysis revealed that the expression of PLA2G1B protein was significantly reduced in the WFC treatment groups (WM and WH) compared to the MG group (*P* < 0.005). These results further confirm that WFC can significantly modulate the expression of PLA2G1B protein. PLA2G1B is an isoform of phosphatase A that releases arachidonic acid and lysophospholipids (Gao et al., 2023; Nevalainen et al., 2000). The expression of PLA2G1B was significantly reduced, resulting in a decrease in arachidonic acid synthesis and subsequent suppression of inflammatory factor expression. PLA2G1B plays a crucial role in modulating immune and inflammatory responses and is involved in the regulation of the NF-kB pathway (Beck et al., 2003; Jo et al., 2004). In a study of acetic acid-induced gastric ulcers in mice, PLA2G1B as one of the key target proteins regulated specific metabolites and was verified by PCR (Ren et al., 2021).

*Eggerthellaceae* and *Erysipelotrichaceae* exhibited a positive correlation with Indole-3-methyl acetate, suggesting that these two microbiota families may play a role in alleviating inflammation through the production of this metabolite. Indole-3-methyl acetate is known for its anti-inflammatory properties, indicating that *Eggerthellaceae* and *Erysipelotrichaceae* may contribute to the modulation of the host’s immune response and inflammation levels via their metabolic products. In addition, *Eggerthellaceae* and *Erysipelotrichaceae* showed negative correlations with lipid metabolites such as Stearoylglycine, FAHFA (18:1(9Z)/9-O-18:0), and N-Stearoyl GABA, suggesting that these microbiota families may influence key lipid metabolic pathways, thereby regulating lipid homeostasis These lipid metabolites are closely related to energy metabolism, membrane stability, and the synthesis of inflammatory mediators. Gut microbiota not only influence the host’s immune response through the production of specific metabolites but also play a critical role in the regulation of key metabolic pathways.

Although this study highlights the therapeutic potential of WFC in treating chronic atrophic gastritis (CAG), there are several limitations. First, the sample size of animal models used in this study is relatively small, which may affect the generalizability of the results. Larger sample sizes are needed in future studies to improve the reliability of the conclusions. Second, untargeted metabolomics has limitations in identifying metabolites from the arachidonic acid family, as these metabolites may be difficult to detect due to their complex structures or low concentrations. Targeted metabolomics may be required for further investigation of these metabolites. Additionally, while the study analyzed correlations between metabolomics data and gut microbiome data, the specific interactions between the microbiota and metabolic changes were not fully elucidated. Further research is needed to explore the causal relationships between these factors.

The findings of this study provide novel insights into the clinical potential of microbiota-targeted therapies for CAG. By demonstrating that WFC restores gut microbial balance and modulates key metabolic pathways—particularly glycerophospholipid and arachidonic acid metabolism—this work highlights a gut microbiota–metabolite axis that may be clinically actionable. The identification of PLA2G1B as a putative microbial-metabolic mediator further suggests a druggable target for future interventions. These results support the rationale for integrating microbiome-modulating agents such as WFC into clinical strategies aimed at improving mucosal repair, reducing chronic inflammation, and preventing progression to gastric cancer in CAG patients. Future clinical trials are warranted to validate these translational implications.

## 5 Conclusion

This study integrated microbiome-metabolome analyses and network pharmacology to uncover the critical role of gut microbiota in the therapeutic action of WFC against CAG. Our results demonstrate that WFC treatment effectively reverses intestinal dysbiosis associated with CAG, significantly modulating the abundance and diversity of four bacterial families and 10 genera. These microbiota alterations were closely correlated with restoration of metabolic pathways, particularly glycerophospholipid and arachidonic acid metabolism, which are known to be microbiota-dependent processes. Furthermore, our analysis suggests PLA2G1B as a potential key target mediating these microbiome-driven metabolic improvements. These findings highlight the central role of gut microbial modulation in the efficacy of WFC, offering novel insights into microbiota-based therapeutic strategies for gastrointestinal disorders.

## Data Availability

The data presented in the study are deposited in NCBI Sequence Read Archive (SRA) repository, accession number PRJNA1311240.

## Footnotes

1
http://www.swissadme.ch/

2
https://old.tcmsp-e.com/tcmsp.php

3
http://swisstargetprediction.ch/

4
https://www.omim.org/

5
https://www.pharmgkb.org/

6
https://www.genecards.org/

7
https://www.disgenet.org/

8
https://cn.string-db.org/cgi/network

9
https://www.metaboanalyst.ca/

10
https://www.rcsb.org/

## References

[B1] Asante I. Pei H. Zhou E. Liu S. Chui D. Yoo E. (2019). Exploratory metabolomic study to identify blood-based biomarkers as a potential screen for colorectal cancer. *Mol. Omics* 15 21–29. 10.1039/c8mo00158h 30515501 PMC6413524

[B2] Beck G. Yard B. A. Schulte J. Haak M. van Ackern K. van der Woude F. J. (2003). Secreted phospholipases A2 induce the expression of chemokines in microvascular endothelium. *Biochem. Biophys. Res. Commun.* 300 731–737. 10.1016/s0006-291x(02)02920-0 12507511

[B3] Bian Y. Chen X. Cao H. Xie D. Zhu M. Yuan N. (2021). A correlational study of Weifuchun and its clinical effect on intestinal flora in precancerous lesions of gastric cancer. *Chin. Med.* 16:120. 10.1186/s13020-021-00529-9 34801051 PMC8605594

[B4] Chen X. Zhang J. Wang R. Liu H. Bao C. Wu S. (2020). UPLC-Q-TOF/MS-Based serum and urine metabonomics study on the ameliorative effects of palmatine on *Helicobacter pylori*-Induced chronic atrophic gastritis. *Front. Pharmacol.* 11:586954. 10.3389/fphar.2020.586954 33041831 PMC7522567

[B5] Chinese Pharmacopoeia Commission (2020). *Chinese pharmacopoeia.* Beijing: China Medical Science and Technology Press.

[B6] Correa P. (1992). Human gastric carcinogenesis: A multistep and multifactorial process–First American Cancer society award lecture on Cancer epidemiology and prevention. *Cancer Res.* 52 6735–6740. 10.1158/0008-5472.CAN-92-0215 1458460

[B7] Fan Y. Pedersen O. (2021). Gut microbiota in human metabolic health and disease. *Nat. Rev. Microbiol.* 19 55–71. 10.1038/s41579-020-0433-9 32887946

[B8] Fan Z. Guo C. Zhang Y. Yao J. Liao L. Dong J. (2019). Hongjingtian injection inhibits proliferation and migration and promotes apoptosis in high glucose-induced vascular smooth muscle cells. *Drug Des. Dev. Ther.* 13 4115–4126. 10.2147/DDDT.S220719 31827318 PMC6901383

[B9] Feng L. Bao T. Bai L. Mu X. Ta N. Bao M. (2023). Mongolian medicine formulae Ruda-6 alleviates indomethacin-induced gastric ulcer by regulating gut microbiome and serum metabolomics in rats. *J. Ethnopharmacol.* 314:116545. 10.1016/j.jep.2023.116545 37196816

[B10] Fung T. C. Olson C. A. Hsiao E. Y. (2017). Interactions between the microbiota, immune and nervous systems in health and disease. *Nat. Neurosci.* 20 145–155. 10.1038/nn.4476 28092661 PMC6960010

[B11] Gao Y. Qian Q. Xun G. Zhang J. Sun S. Liu X. (2023). Integrated metabolomics and network analysis reveal changes in lipid metabolisms of tripterygium glycosides tablets in rats with collagen-induced arthritis. *Comput. Struct. Biotechnol. J.* 21 1828–1842. 10.1016/j.csbj.2023.02.050 36923473 PMC10009339

[B12] Guan W. L. He Y. Xu R. H. (2023). Gastric cancer treatment: Recent progress and future perspectives. *J. Hematol. Oncol.* 16:57. 10.1186/s13045-023-01451-3 37245017 PMC10225110

[B13] Guo W. L. Cao Y. J. You S. Z. Wu Q. Zhang F. Han J. Z. (2022). Ganoderic acids-rich ethanol extract from *Ganoderma lucidum* protects against alcoholic liver injury and modulates intestinal microbiota in mice with excessive alcohol intake. *Curr. Res. Food. Sci.* 5 515–530. 10.1016/j.crfs.2022.02.013 35281335 PMC8913248

[B14] Huang M. Li S. He Y. Lin C. Sun Y. Li M. (2021). Modulation of gastrointestinal bacterial in chronic atrophic gastritis model rats by Chinese and west medicine intervention. *Microb. Cell Fact.* 20:31. 10.1186/s12934-021-01525-2 33530970 PMC7852297

[B15] Huang W. Zhu J. Wang Y. Gan H. Yang Z. (2022). Effect of electroacupuncture at zusanli (ST36) on gut microbiota in rats with chronic atrophic gastritis. *Front. Genet.* 13:824739. 10.3389/fgene.2022.824739 35281809 PMC8906781

[B16] Jian C. Wei L. Wu T. Li S. Wang T. Chen J. (2023). Comprehensive multi-omics analysis reveals the core role of glycerophospholipid metabolism in rheumatoid arthritis development. *Arthritis Res. Ther.* 25:246. 10.1186/s13075-023-03208-2 38102690 PMC10722724

[B17] Jo E. J. Lee H. Y. Lee Y. N. Kim J. I. Kang H. K. Park D. W. (2004). Group IB secretory phospholipase A2 stimulates CXC chemokine ligand 8 production via ERK and NF-kappa B in human neutrophils. *J. Immunol.* 173 6433–6439. 10.4049/jimmunol.173.10.6433 15528384

[B18] Kau A. L. Ahern P. P. Griffin N. W. Goodman A. L. Gordon J. I. (2011). Human nutrition, the gut microbiome and the immune system. *Nature* 474 327–336. 10.1038/nature10213 21677749 PMC3298082

[B19] Kim E. J. Hong Y. S. Seo S. H. Park S. E. Na C. S. Son H. S. (2019). Metabolite markers for characterizing sasang constitution type through GC-MS and (1)H NMR-Based metabolomics study. *Evid. Based Compl. Alternat. Med.* 2019:8783496. 10.1155/2019/8783496 30854017 PMC6378031

[B20] Lee S. Y. Jeong J. J. Eun S. H. Kim D. H. (2015). Anti-inflammatory effects of ginsenoside Rg1 and its metabolites ginsenoside Rh1 and 20(S)-protopanaxatriol in mice with TNBS-induced colitis. *Eur. J. Pharmacol.* 762 333–343. 10.1016/j.ejphar.2015.06.011 26054809

[B21] Li X. Dong H. Chen L. Wang Y. Hao Z. Zhang Y. (2022a). Identification of N7-methylguanosine related subtypes and construction of prognostic model in gastric cancer. *Front. Immunol.* 13:984149. 10.3389/fimmu.2022.984149 36300121 PMC9589367

[B22] Li X. He F. Tuo X. Qiu Y. Guo J. Wu Y. (2022b). Electroacupuncture ameliorates peptic ulcer disease in association with gastroduodenal microbiota modulation in mice. *Front. Cell Infect. Microbiol.* 12:935681. 10.3389/fcimb.2022.935681 36061878 PMC9437313

[B23] Liu H. C. Xiang Z. B. Wang Q. Li B. Y. Jin Y. S. Chen H. S. (2017). Monomeric and dimeric ent-kauranoid-type diterpenoids from rabdosia japonica and their cytotoxicity and anti-HBV activities. *Fitoterapia* 118 94–100. 10.1016/j.fitote.2017.03.006 28300699

[B24] Liu Y. Li H. Wang X. Huang J. Zhao D. Tan Y. (2023). Anti-Alzheimers molecular mechanism of icariin: Insights from gut microbiota, metabolomics, and network pharmacology. *J. Transl. Med.* 21:277. 10.1186/s12967-023-04137-z 37095548 PMC10124026

[B25] Ma C. He J. Lai L. Chen Y. Xue W. Chen J. (2021). Intestinal microbiome and metabolome analyses reveal metabolic disorders in the early stage of renal transplantation. *Mol. Omics* 17 985–996. 10.1039/d1mo00279a 34676841

[B26] Maier J. A. Hla T. Maciag T. (1990). Cyclooxygenase is an immediate-early gene induced by interleukin-1 in human endothelial cells. *J. Biol. Chem.* 265 10805–10808. 10.1016/s0021-9258(19)38515-1 1694171

[B27] Nevalainen T. J. Haapamäki M. M. Grönroos J. M. (2000). Roles of secretory phospholipases A(2) in inflammatory diseases and trauma. *Biochim. Biophys. Acta* 1488 83–90. 10.1016/s1388-1981(00)00112-8 11080679

[B28] Nicholson J. K. Holmes E. Wilson I. D. (2005). Gut microorganisms, mammalian metabolism and personalized health care. *Nat. Rev. Microbiol.* 3 431–438. 10.1038/nrmicro1152 15821725

[B29] Pan X. T. Tao H. Y. Nie M. J. Liu Y. J. Huang P. Liu S. L. (2020). A clinical study of traditional Chinese medicine prolonging the survival of advanced gastric cancer patients by regulating the immunosuppressive cell population: A study protocol for a multicenter, randomized controlled trail. *Medicine* 99:e19757. 10.1097/MD.00000000000019757 32311976 PMC7220101

[B30] Pozuelo M. Panda S. Santiago A. Mendez S. Accarino A. Santos J. (2015). Reduction of butyrate- and methane-producing microorganisms in patients with irritable bowel syndrome. *Sci. Rep.* 5:12693. 10.1038/srep12693 26239401 PMC4523847

[B31] Ren S. Wei Y. Niu M. Li R. Wang R. Wei S. (2021). Mechanism of rutaecarpine on ethanol-induced acute gastric ulcer using integrated metabolomics and network pharmacology. *Biomed. Pharmacother.* 138:111490. 10.1016/j.biopha.2021.111490 33773465

[B32] Reyes-Farias M. Carrasco-Pozo C. (2019). The anti-cancer effect of quercetin: Molecular implications in cancer metabolism. *Int. J. Mol. Sci.* 20:177. 10.3390/ijms20133177 31261749 PMC6651418

[B33] Sheng C. Guo Y. Ma J. Hong E. K. Zhang B. Yang Y. (2022). Metabolomic profiling reveals protective effects and mechanisms of sea buckthorn sterol against carbon tetrachloride-induced acute liver injury in rats. *Molecules* 27:2224. 10.3390/molecules27072224 35408620 PMC9000363

[B34] Stojanov S. Berlec A. Štrukelj B. (2020). The influence of probiotics on the firmicutes/bacteroidetes ratio in the treatment of obesity and inflammatory bowel disease. *Microorganisms* 8:1715. 10.3390/microorganisms8111715 33139627 PMC7692443

[B35] Su X. L. Wang J. W. Che H. Wang C. F. Jiang H. Lei X. (2020). Clinical application and mechanism of traditional Chinese medicine in treatment of lung cancer. *Chin. Med. J.* 133 2987–2997. 10.1097/CM9.000000000000001141 33065603 PMC7752681

[B36] Sung H. Ferlay J. Siegel R. L. Laversanne M. Soerjomataram I. Jemal A. (2021). Global Cancer statistics 2020: GLOBOCAN estimates of incidence and mortality worldwide for 36 cancers in 185 countries. *CA Cancer J. Clin.* 71 209–249. 10.3322/caac.21660 33538338

[B37] Sung J. J. Leung W. K. Go M. Y. To K. F. Cheng A. S. Ng E. K. (2000). Cyclooxygenase-2 expression in Helicobacter pylori-associated premalignant and malignant gastric lesions. *Am. J. Pathol.* 157 729–735. 10.1016/S0002-9440(10)64586-5 10980112 PMC1885697

[B38] Tian T. Mao Q. Xie J. Wang Y. Shao W. H. Zhong Q. (2022). Multi-omics data reveals the disturbance of glycerophospholipid metabolism caused by disordered gut microbiota in depressed mice. *J. Adv. Res.* 39 135–145. 10.1016/j.jare.2021.10.002 35777903 PMC9263645

[B39] Tong Y. Jing M. Zhao X. Liu H. Wei S. Li R. (2021). Metabolomic study of zuojin pill in relieving 1-methyl-3-nitro-1-nitrosoguanidine-induced chronic atrophic gastritis. *Evid. Based Compl. Alternat. Med.* 2021:7004798. 10.1155/2021/7004798 34956382 PMC8709764

[B40] Wang B. Wu L. Chen J. Dong L. Chen C. Wen Z. (2021a). Metabolism pathways of arachidonic acids: Mechanisms and potential therapeutic targets. *Signal Transduct. Target Ther.* 6:94. 10.1038/s41392-020-00443-w 33637672 PMC7910446

[B41] Wang R. Li S. Jia H. Si X. Lei Y. Lyu J. (2021b). Protective effects of cinnamaldehyde on the inflammatory response, oxidative stress, and apoptosis in liver of salmonella typhimurium-challenged mice. *Molecules* 26:2309. 10.3390/molecules26082309 33923441 PMC8073330

[B42] Wang B. Zhou W. Zhang H. Wang W. Zhang B. Li S. (2023a). Exploring the effect of Weifuchun capsule on the toll-like receptor pathway mediated HES6 and immune regulation against chronic atrophic gastritis. *J. Ethnopharmacol.* 303:115930. 10.1016/j.jep.2022.115930 36403744

[B43] Wang Y. He Y. Liang Y. Liu H. Chen X. Kulyar M. F. (2023b). Fecal microbiota transplantation attenuates *Escherichia coli* infected outgrowth by modulating the intestinal microbiome. *Microb. Cell Fact* 22:30. 10.1186/s12934-023-02027-z 36803386 PMC9936653

[B44] Wang Z. Wu W. Guan X. Guo S. Li C. Niu R. (2020). 20(S)-Protopanaxatriol promotes the binding of P53 and DNA to regulate the antitumor network via multiomic analysis. *Acta Pharm. Sin. B* 10 1020–1035. 10.1016/j.apsb.2020.01.017 32642409 PMC7332671

[B45] Wu Z. Zhang L. Xue N. Sun W. Zhou X. Fang M. (2022). Structure of intestinal flora in rats with chronic gastritis induced by deoxycholic acid. *Chin. J. Microecol.* 34 125–133. 10.13381/j.cnki.cjm.202202001

[B46] Xi Z. Wang M. Xia J. Li H. Hua Y. Xu T. (2021). Explore the effects of Shidan granules on chronic atrophic gastritis using LC-MS based plasma metabolomics study. *Biomed. Chromatogr.* 35:e5129. 10.1002/bmc.5129 33780017

[B47] Xie D. Wu C. Wang D. Nisma Lena B. A. Liu N. Ye G. (2024). Wei-fu-chun tablet halted gastric intestinal metaplasia and dysplasia associated with inflammation by regulating the NF-κB pathway. *J. Ethnopharmacol.* 318:117020. 10.1016/j.jep.2023.117020 37567428

[B48] Yang X. Chi C. Li W. Zhang Y. Yang S. Xu R. (2024). Metabolomics and lipidomics combined with serum pharmacochemistry uncover the potential mechanism of Huang-Lian-Jie-Du decoction alleviates atherosclerosis in ApoE(-/-) mice. *J. Ethnopharmacol.* 324:117748. 10.1016/j.jep.2024.117748 38216103

[B49] Yu C. Su Z. Li Y. Li Y. Liu K. Chu F. (2020). Dysbiosis of gut microbiota is associated with gastric carcinogenesis in rats. *Biomed. Pharmacother.* 126:110036. 10.1016/j.biopha.2020.110036 32172061

[B50] Yuan Z. Yang L. Zhang X. Ji P. Hua Y. Wei Y. (2020). Mechanism of Huang-lian-Jie-du decoction and its effective fraction in alleviating acute ulcerative colitis in mice: Regulating arachidonic acid metabolism and glycerophospholipid metabolism. *J. Ethnopharmacol.* 259:112872. 10.1016/j.jep.2020.112872 32417423

[B51] Zhou P. Yang T. Xu M. Zhao Y. Shen P. Wang Y. (2022). 16S rRNA sequencing-based evaluation of the protective effects of Hua-Zhuo-Jie-Du on rats with chronic atrophic gastritis. *BMC Compl. Med. Ther.* 22:71. 10.1186/s12906-022-03542-z 35296316 PMC8928654

[B52] Zou Y. T. Zhou J. Wu C. Y. Zhang W. Shen H. Xu J. D. (2021). Protective effects of *Poria cocos* and its components against cisplatin-induced intestinal injury. *J. Ethnopharmacol.* 269:113722. 10.1016/j.jep.2020.113722 33352240

