# Supplementary material for: Gut microbiome modulation by Weifuchun Capsules alleviates chronic atrophic gastritis: a combined microbiota and metabolomics approach
Source: Front Microbiol. 2025 Sep 2;16:1634410. doi: 10.3389/fmicb.2025.1634410 (PMC12436308; doi:10.3389/fmicb.2025.1634410)
Supplement: Supplementary file 2 [file Table_2.DOCX]

Table S1

Identification of Compounds by UHPLC-Q-TOF/MS.

| No. | Identification | Formula | Adducts | M/Z | Error (ppm) | t_R_ (min) | MS/MS fragments |
| --- | --- | --- | --- | --- | --- | --- | --- |
| 1 | Sucrose | C_12_H_22_O_11_ | -H | 341.1089 | -0.2 | 0.87 | 341.1116,179.0508,161.0480,149.0683 |
| 2 | Citric acid | C_6_H_8_O_7_ | -H | 191.0204 | 3.7 | 1.14 | 191.0215,130.0464,129.0212,111.0108,87.0112,85.0321 |
| 3 | Syringic acid | C_9_H_10_O_5_ | -H | 197.0460 | 2.2 | 2.00 | 197.0474,179.0366,135.0470,123.0473,72.9962 |
| 4 | Chlorogenic acid | C_16_H_18_O_9_ | -H | 353.0872 | -1.7 | 3.13 | 353.0898,191.0585,179.0375,173.0487,161.0274,135.0470,85.0322 |
| 5 | Caffeic acid | C_9_H_8_O_4_ | -H | 179.0363 | 7.4 | 3.26 | 179.0349,135.0469,134.0385,107.0516,89.0426 |
| 6 | Scopoletin | C_10_H_8_O_4_ | -H | 191.0353 | 1.7 | 4.52 | 191.0339,176.0133 |
| 7 | Ferulic acid | C_10_H_10_O_4_ | -H | 193.0515 | 4.5 | 4.59 | 193.0515,178.0280,134.0392 |
| 8 | 4-formylphenoxyacetic acid | C_9_H_8_O_4_ | -H | 179.0353 | 1.6 | 4.66 | 179.0368,135.0464,107.0520 |
| 9 | Vicenin 2 | C_27_H_30_O_15_ | -H | 593.1504 | -1.4 | 5.03 | 593.1537,575.1447,503.1214,473.1098,413.0894,383.0782,353.0673,325.0738 |

| 10 | Blechnic acid | C_18_H_14_O_8_ | -H | 357.0622 | 1.6 | 5.26 | 357.0651,313.0747,269.0845,,203.0375,159.0472,109.0315 |
| --- | --- | --- | --- | --- | --- | --- | --- |
| 11 | Lonicerin | C_27_H_30_O_15_ | -H | 593.1511 | -0.2 | 5.33 | 593.1557,473.1126,383.0804,353.0693 |
| 12 | Narirutin-4'-glucoside | C_33_H_42_O_19_ | -H | 741.2265 | 2.4 | 5.48 | 741.2338,579.1776,459.1173,433.1152,271.0640,151.0050 |
| 13 | Naringenin-7-O-glucuronide | C_21_H_20_O_11_ | -H | 447.0952 | 4.3 | 5.69 | 447.0969,327.0536,285.0439,284.0358 |
| 14 | Eriocitrin | C_27_H_32_O_15_ | -H | 595.1672 | 0.7 | 5.98 | 595.1709,287.0586,151.0057 |
| 15 | Eriodictyol | C_15_H_12_O_6_ | -H | 287.0577 | 5.6 | 6.04 | 287.0594,243.0327,151.0055,135.0471 |
| 16 | Rutin | C_27_H_30_O_16_ | -H | 609.1475 | 2.3 | 6.11 | 609.1505,301.0366,300.0292,271.0268,255.0319,151.0051 |
| 17 | Enmein* | C_20_H_26_O_6_ | -H | 361.1664 | 2.0 | 6.23 | 361.1690,343.1587,331.1585 |
| 18 | Quercitrin | C_21_H_20_O_11_ | -H | 447.0934 | 6.7 | 6.46 | 447.0964,285.0437 |
| 19 | Trichorabdonin | C_20_H_28_O_7_ | -H | 379.1761 | -0.3 | 6.68 | 379.1807,361.1712,343.1601,333.1792,299.1693,281.1564,271.1725 |
| 20 | Narirutin | C_27_H_32_O_14_ | -H | 579.1766 | 2.7 | 6.85 | 579.1766,271.0634,151.0056 |
| 21 | Nervosanin B* | C_20_H_30_O_6_ | -H | 365.1973 | 1.0 | 7.03 | 365.1979,347.1885,335.1885,329.1844,317.1789,299.1688,281.1558 |
| 22 | Neoeriocitrin | C_27_H_32_O_15_ | -H | 595.1678 | 1.6 | 7.10 | 595.1711,475.1156,287.0589,271.0628,151.0057 |
| 23 | Rhoifolin | C_27_H_30_O_14_ | -H | 577.1573 | 1.7 | 7.22 | 577.1599,431.1025,413.0898,269.0473,151.0045 |
| 24 | p-hydroxybenzoic acid | C_7_H_6_O_3_ | -H | 137.0251 | 4.8 | 7.29 | 137.0251,93.0364,65.0435 |
| 25 | Diosmin | C_28_H_32_O_15_ | -H | 607.1679 | 1.7 | 7.40 | 607.1734,299.0590,284.0354 |

| 26 | Hesperidin | C_28_H_34_O_15_ | -H | 609.1828 | 0.5 | 7.43 | 609.1867,301.0736,286.0509,242.0607,151.0052 |
| --- | --- | --- | --- | --- | --- | --- | --- |
| 27 | Rosmarinic acid | C_18_H_16_O_8_ | -H | 359.0781 | 2.4 | 7.54 | 359.0788,197.04659,179.0361,161.0258,135.0463 |
| 28 | Neodiosmin | C_28_H_32_O_15_ | -H | 607.1676 | 1.2 | 7.64 | 607.1710,299.0587,284.0352 |
| 29 | Rabdoternin B | C_20_H_28_O_7_ | -H | 379.1751 | -2.9 | 7.68 | 379.1788,361.1661,343.1584,333.1731,317.1786,299,1681,281.1565 |
| 30 | Neohesperidin | C_28_H_34_O_15_ | -H | 609.1836 | 1.9 | 7.71 | 609.1848,489.1428,343.0842,301.0730,286.0499,242.0603,151.0055 |
| 31 | Notoginsenoside N | C_48_H_82_O_19_ | -H | 961.5424 | 4.8 | 8.06 | 961.5678,799.4925,781.4859637.4329,475.3765 |
| 32 | Naringin* | C_27_H_32_O_14_ | -H | 579.1733 | 2.4 | 8.16 | 579.1764,459.1193,271.0637,151.0059 |
| 33 | Notoginsenoside R1 | C_47_H_80_O_18_ | -H | 931.5308 | 0.6 | 8.32 | 931.5379,799.4965,637.4384,475.3827 |
| 34 | Isorhoifolin | C_27_H_30_O_14_ | -H | 577.1553 | -1.7 | 8.53 | 577.1599,269.0414,151.0051 |
| 35 | Epinodosinol* | C_20_H_28_O_6_ | -H | 363.1808 | -1.5 | 8.65 | 363.1831,345.1740,333.1726,327.1603,319.1939,315.1623,301.1832.283.1725 |
| 36 | Ginsenoside Re* | C_48_H_82_O_18_ | -H | 945.5462 | 3.6 | 8.82 | 945.5494,799.4924,783.4963,637.4369,619.4268,475.3822,161.0476 |
| 37 | Ginsenoside Rg1* | C_42_H_72_O_14_ | -H | 845.4927 | 4.0 | 8.84 | 845.4957,799.4907,637.4320,547.5583,475.3808,391.2806,179.0583,161.0478 |
| 38 | 6'-acetyl-Rg1 | C_44_H_74_O_15_ | -H | 841.4969 | 1.7 | 9.39 | 841.5023,799.4844,781.4848,637.4379,619.4243,475.3845,161.0477 |
| 39 | Acacetin | C_16_H_12_O_5_ | -H | 283.0621 | 3.1 | 9.44 | 283.0630,268.0394,239.0373,151.0069 |

| 40 | Luteolin | C_15_H_10_O_6_ | -H | 285.0404 | -0.3 | 9.48 | 285.0434,243.0308,241.0508,217.0526,151.0053,133.0317 |
| --- | --- | --- | --- | --- | --- | --- | --- |
| 41 | 1α,6β,7α-trihydroxy-7α,20-epoxykaur-9(11),16-dien-15-one | C_20_H_26_O_5_ | -H | 345.1715 | 2.2 | 9.77 | 345.1743,327.1660,301.1848,297.1499,283.1738 |
| 42 | Oxypeucedanin | C_16_H_14_O_5_ | -H | 285.0774 | 2.0 | 9.97 | 285.0792,270.0556,243.0691,175.0779,164.0131,151.0058,136.0188,108.0227 |
| 43 | Epinodosin* | C_20_H_26_O_6_ | -H | 361.1648 | -2.4 | 10.37 | 361.1654,331.1537,317.1777,299.1671,287.1708 |
| 44 | Lasiodonin* | C_20_H_28_O_6_ | -H | 363.1814 | 0.3 | 10.69 | 363.1835,345.1743,333.1741,319.1930,315.1610 |
| 45 | Enanderianin C | C_22_H_30_O_7_ | -H | 405.1931 | 3.1 | 10.78 | 405.1952.363.1848,345.1740,301.1825,283.1729 |
| 46 | Naringenin | C_15_H_12_O_5_ | -H | 271.0619 | 2.4 | 10.80 | 271.0639,187.0431,151.0059,119.0527,107.0164 |
| 47 | Rubescensin S | C_20_H_28_O_7_ | -H | 379.1776 | 3.5 | 10.82 | 379.1795,361.1666,343.1545,333.1729,317.1784,299.1679,281.1551,271.1722,255.1782 |
| 48 | Oridonin* | C_20_H_28_O_6_ | -H | 363.1808 | -1.3 | 10.86 | 363.1836,345.1736,333.1714,327.1632,315.1641,299.1681,297.1474,283.1723,271.1717 |
| 49 | Glaucocalyxin A* | C_20_H_28_O_4_ | -H | 331.1926 | 3.4 | 10.88 | 331.1940,313.1836,285.1877,269.1928,149.0623 |
| 50 | Umbelliferone | C_9_H_6_O_3_ | -H | 161.0258 | 8.5 | 10.96 | 161.0271,133.0317,105.0360 |
| 51 | Scoparone | C_11_H_10_O_4_ | -H | 205.0522 | 7.4 | 10.99 | 205.0523,161.0261 |
| 52 | Diosmetin | C_16_H_12_O_6_ | -H | 299.0571 | 3.3 | 11.01 | 299.0593,284.0348,227.0371,136.9899 |

| 53 | Apigenin | C_15_H_10_O_5_ | -H | 269.0472 | 6.2 | 11.09 | 269.0477,225.0571,227.0385,201.0574,183.0456151.0051,117.0355 |
| --- | --- | --- | --- | --- | --- | --- | --- |
| 54 | Umbrosin A | C_20_H_30_O_4_ | -H | 333.2073 | 0.6 | 11.42 | 333.2091,315.1955,297.1918,287.2063,271.1707 |
| 55 | Ponicidin* | C_20_H_26_O_6_ | -H | 361.1666 | 2.5 | 11.44 | 361.1678,343.1564,317.1782,299.1682,271.1735.255.1781,253.1625 |
| 56 | Sodoponin | C_22_H_32_O_7_ | -H | 407.2082 | 1.6 | 11.46 | 407.2091,365.1987,347.1881,329.1769,317.1776 |
| 57 | Hesperitin | C_16_H_14_O_6_ | -H | 301.0711 | -2.2 | 11.52 | 301.0737,286.0506,257.0849,242.0607,164.0138;151.0159,134.0384,108.0243 |
| 58 | Ginsenoside Rf* | C_42_H_72_O_14_ | -H | 799.4875 | 3.4 | 11.71 | 799.4925,637.4375,475.3835,391.2887,221.0689,161.0478 |
| 59 | Notoginsenoside Fa | C_59_H_100_O_27_ | -H | 1239.6402 | 1.8 | 11.81 | 1239.6526,945.5366,621.4331,353.1071 |
| 60 | Rabdosinatol | C_20_H_30_O_4_ | -H | 333.2071 | -0.1 | 12.21 | 333.2100,315.1981,297.1917,287.206 |
| 61 | Notoginsenoside R2 | C_41_H_70_O_13_ | -H | 769.4769 | 3.2 | 12.22 | 769.4789,637.4360,619.4209,475.3819,391.2874 |
| 62 | Rabdoternin A* | C_20_H_28_O_6_ | -H | 363.1811 | -0.5 | 12.30 | 363.1843,345.1739,333.1740,327.1631,315.1626,301.1836,283.1727 |
| 63 | Lasiokaurinol* | C_22_H_32_O_7_ | -H | 407.2079 | 0.9 | 12.40 | 407.2109,365.1992,347.1894,329.1832,317.1806,299.1690 |
| 64 | Ginsenoside Ra1/Ra2 | C_58_H_98_O_26_ | -H | 1209.6311 | 3.1 | 12.51 | 1209.6383,1077.6017,945.5844,783.4945,621.4469,459.3899,323.1005 |
| 65 | Xerophilusin B | C_20_H_26_O_5_ | -H | 345.1719 | 3.3 | 12.67 | 345.1740,327.1621,309.1491,301.1858,299.1603 |

| 66 | Amethystonoic acid | C_20_H_28_O_6_ | -H | 363.1815 | 0.4 | 12.69 | 363.1845,345.1743,327.1633,301.1837,283.1732 |
| --- | --- | --- | --- | --- | --- | --- | --- |
| 67 | Ginsenoside Rb1* | C_54_H_92_O_23_ | -H | 1107.5997 | 3.6 | 12.71 | 1107.6045,945.5526,783.4983,621.4442,459.3806,323.0997,221.0689,179.0584,161.0480 |
| 68 | Taibaihenryiin A | C_22_H_30_O_7_ | -H | 405.1923 | 1.0 | 12.79 | 405.1957,363.1847,345.1745,315.1648 |
| 69 | Nodosin* | C_20_H_26_O_6_ | -H | 361.1664 | 2.1 | 12.81 | 361.1677,343.1546,333.1745,317.1795,299.1683,287.1641 |
| 70 | Ginsenoside Rh1 | C_36_H_62_O_9_ | -H | 637.4328 | 1.1 | 12.85 | 637.4364,475.3819,161.0465 |
| 71 | Enmenol* | C_20_H_30_O_6_ | -H | 365.1978 | 2.3 | 12.87 | 365.2001,347.1899,329.1785,317.1775,311.1693,299.1669 |
| 72 | 6-acetyl-Rb1 | C_56_H_94_O_24_ | -H | 1149.6101 | 3.4 | 12.88 | 1149.6191,1107.6027,1089.6000,945.5525,783.5001,621.4476,459.3853 |
| 73 | Ginsenoside Rg2 | C_42_H_72_O_13_ | -H | 783.4949 | 6.2 | 12.89 | 783.4966,637.4375,619.4196,475.3808,391.2942,161.0471 |
| 74 | Lasiokaurin* | C_22_H_30_O_7_ | -H | 405.1921 | 0.5 | 12.97 | 405.1951,387.1808,363.1839,345.1729,315.1631,299.1689 |
| 75 | Ginsenoside Rb2* | C_53_H_90_O_22_ | -H | 1077.5883 | 3.0 | 13.07 | 1077.5926,945.5540,915.5437,783.4977,621.4442,459.3877,191.0584,149.0480 |
| 76 | 20(R)-Ginsenoside Rh1 | C_36_H_62_O_9_ | -H | 637.4331 | 1.6 | 13.14 | 637.4325,475.3829, |
| 77 | Ginsenoside Ro | C_48_H_76_O_19_ | -H | 955.4934 | -4.9 | 13.17 | 955.4964,793.4447,731.4451,613.3794,569.3901,523.3834,455.3489 |
| 78 | Ginsenoside Rs1/Rs2 | C_55_H_92_O_23_ | -H | 1119.5973 | 1.4 | 13.33 | 1119.6086,1077.5986,945.5487,783.5096,621.4318,459.3846,149.0466 |

| 79 | Ginsenoside Rb3 | C_53_H_90_O_22_ | -H | 1077.5879 | 2.6 | 13.45 | 1077.5957,945.5506,915.5412,783.4973,621.4416,459.3894,191.0581,149.0470 |
| --- | --- | --- | --- | --- | --- | --- | --- |
| 80 | Ginsenoside Rc | C_53_H_90_O_22_ | -H | 1077.5870 | 1.8 | 13.59 | 1077.5943,945.5458,915.5380,783.5019,621.4502,459.4020 |
| 81 | 7α,14β-7,14-dihydroxykaur-16-ene-3,11,15-trione | C_20_H_26_O_5_ | -H | 345.1701 | -1.8 | 14.14 | 345.1723,327.1666,301.1826,283.1708 |
| 82 | Limonin | C_26_H_30_O_8_ | -H | 469.1880 | 2.6 | 14.25 | 469.1888,425.1970,381.2105,321.1166,306.1287,283.0999,278.1338,229.1259,199.1150 |
| 83 | Gypenoside XⅦ | C_48_H_82_O_18_ | -H | 945.5456 | 2.9 | 14.32 | 945.5506,783.4996,765.4881,621.4430,603.4292,459.3902,179.0595,161.0485 |
| 84 | Chikusetsusaponin Iva | C_42_H_66_O_14_ | -H | 793.4412 | 4.1 | 14.39 | 793.4452,631.3916,613.3782,587.4056,569.3895,455.3547 |
| 85 | 5,6-dihydroxy-7,4'-dimethoxy-flavone | C_17_H_14_O_6_ | -H | 313.0737 | 6.1 | 14.40 | 313.0748,298.0515,283.0276,161.0061 |
| 86 | 6'-acetyl-Rd | C_50_H_84_O_19_ | -H | 987.5552 | 1.8 | 14.53 | 987.5641,945.5551,927.5443,783.5017,765.4886,621.4425,459.3814 ,161.0482 |
| 87 | Nomilinic acid | C_28_H_36_O_10_ | -H | 531.2236 | 0.1 | 14.86 | 531.2273,489.2165,471.2057,427.2160,369.1734,325.1837,307.1730,273.1502 |
| 88 | Notoginsenoside K | C_48_H_82_O_18_ | -H | 945.5440 | 1.3 | 15.03 | 945.5522,783.4963,621.4507,459.3998,323.0985,221.0664,179.0580,119.0375 |
| 89 | Heraclenin | C_16_H_14_O_5_ | -H | 285.0788 | 6.7 | 15.28 | 285.0796,270.0563,243.0684,175.0777 |

| 90 | 7α,10α,14β-10,14,18-tiihydroxykaura-11.16-dien-15-one | C_20_H_28_O_4_ | -H | 331.1925 | 3.2 | 15.30 | 331.1949,313.1818,285.1876,269.1933 |
| --- | --- | --- | --- | --- | --- | --- | --- |
| 91 | Ginsenoside Rd2 | C_47_H_80_O_17_ | -H | 915.5352 | 3.2 | 15.72 | 915.5426,783.4958,753.4794,621.4449,459.3886,161.0466 |
| 92 | Ginsenoside Rd* | C_48_H_82_O_18_ | -H | 945.5477 | 5.2 | 15.74 | 945.5522,783.5006,765.4878,621.4357,459.3899,365.1784 |
| 93 | NotoginsenosideFe/GynosaponinⅠ/VinaginsenosideR16/R17 | C_47_H_80_O_17_ | -H | 915.5331 | 0.9 | 15.83 | 915.5415,783.5027,753.4727,621.4410 |
| 94 | Ginsenoside Rg6/F4 | C_42_H_70_O_12_ | -H | 765.4822 | 3.6 | 16.73 | 765.4885,619.4264,457.3743 |
| 95 | SpinasaponinA | C_42_H_66_O_14_ | -H | 793.4407 | 3.5 | 17.38 | 793.4453,731.4494,631.3915,613.3809,587.3991,569.3905,455.3550 |
| 96 | Rabdoumbrosanin | C_20_H_28_O_3_ | -H | 315.1965 | -0.3 | 17.73 | 315.1995,297.1913,287.2043 |
| 97 | Ginsenoside Rg3* | C_42_H_72_O_13_ | -H | 783.4958 | 7.4 | 17.84 | 783.4958,621.4438,459.3871,375.2967 |
| 98 | 20(S)-6"-O-acetylginsenoside Rg3/20(R)-6"-O-acetylginsenoside Rg3 | C_44_H_74_O_14_ | -H | 825.5022 | 2.0 | 17.86 | 825.5080,783.4971,765.4877,663.4486,621.4409,459.3873,161.0480,101.0262,59.0176 |
| 99 | Gingerglycolipid B | C_33_H_58_O_14_ | -H | 677.3769 | 2.3 | 18.41 | 677.3821,609.4160,415.1493,397.1379,279.2351,235.0842,161.0490,89.0274 |

* Compound identified by comparison with a reference standard

Table S2

Drugs with a common target.

| ID | Compound | Target |
| --- | --- | --- |
| RG1 | DNOP | PRKCA,OXTR,ABL1,CRHR1,NOD2,PDE5A,MAPK14,PDGFRB,PTGS2,CYP2C9,KRAS,CASP3,CASP1 |
| A | β-sitosterol | AR,CYP2C19,G6PD,VDR,NOS2,PPARG |
| RG2 | PalmitoleicAcid | PPARG,TERT,PTGS1 |
| RG3 | Protopanaxatriol | CRHR1,PDE5A,MAPK14,PTGS2,CYP2C9,AR,CYP2C19,VDR,PTGS1,ATP12A,PIK3CB,PIK3CD,PIK3CG,PIK3CA,MAPK8,CYP3A4,MTOR,ITGAL,STAT3,ERBB2,JAK1,IL6ST,ADORA2B,MDM2,KDR |
| RG4 | Protopanaxadiol | MAPK14,PTGS2,CYP2C9,AR,CYP2C19,VDR,PTGS1,ATP12A,PIK3CB,PIK3CD,PIK3CG,PIK3CA,MAPK8,CYP3A4,IL6ST,MDM2,KDR |
| IA1 | (+)-1-Hydroxypinoresinol | PIK3CG,PIK3CA,ALOX5,HIF1A,MCL1 |
| IA2 | 6, 7-Dehydroroyleanone | MDM2,MCL1,BCL2 |
| IA3 | 7-α-Hydroxystigmasterol | CRHR1,MAPK14,CYP2C9,AR,CYP2C19,VDR,NOS2,PPARG,MAPK8,CYP3A4,ITGAL,MDM2,KDR,CHRM3,CCND1,ICAM1,ITGB2,CCKBR,PRKCB,CXCR3 |
| IA4 | Amethystonoic acid | PTGS2,AR,MAPK8,AKT1,SELP,SELL,SELE,MMP12,CTNNB1,PARP1 |
| B | Caffeic acid | ALOX5,MMP9,MMP1,MMP2 |

| IA5 | Angustifolin | PTGS2,CASP1,AR,PIK3CA,JAK1,MDM2,AKT1,PARP1,TLR9,TYK2,MPO,HMOX1,MAPK1,CTRC,NTRK1,CASP8,VCAM1 |
| --- | --- | --- |
| IA6 | Cirsiliol | PTGS2,NOS2,TERT,KDR,MMP12,PARP1,ALOX5,MMP9,MMP2,MPO,EGFR,MMP3,F2,MET,CD38,SRC,SYK,MCL1,BCL2,PLA2G1B,XDH,AHR,TTR,CFTR,BCL2L1 |
| IA7 | Coetsoidin A | MAPK14,CASP3,CASP1,TERT,JAK1,IL6ST,MDM2,KDR,AKT1,PARP1,MMP1,TYK2,CHRM3,EGFR,MAPK1,CXCR2,NTRK1,SRC,CASP8,TNF,HRH2 |
| IA8 | Daucosterol | STAT3,IL2 |
| IA9 | dibutyl phthalate | PRKCA,CRHR1,MAPK14,PTGS2,CYP2C9,CASP3,CYP2C19,PTGS1,MAPK8,CYP3A4,ADORA2B,MIF,F2,CTRC,CXCR2,ADAM17,TNFRSF1A,MEN1,ALDH2 |
| IA10 | Dibutyl terephthalate | PRKCA,MAPK14,CASP3,CASP1,AR,JAK1,SELE,TYK2,HDAC6,VCAM1,MEN1,ICAM1,HPGD |
| IA11 | Enmenol | STAT3,TLR9,IL2 |
| IA12 | Epipinoresinol | PIK3CG,PIK3CA,ALOX5,HIF1A,MCL1 |
| IA13 | Esculetin | EGFR,XDH |
| IA14 | Glaucocalyxin A | MAPK14,PTGS2,CYP2C9,AR,JAK1,MDM2,KDR,PARP1,MMP9,MMP1,MMP2,TYK2,MPO,CHRM3,HMOX1,MMP8,ADA,CCR5,EGFR,MIF |
| IA15 | Glaucocalyxin B | MAPK14,PTGS2,CYP2C9,AR,MAPK8,MTOR,JAK1,IL6ST,MDM2,PARP1,ALOX5,MMP9,MMP1,TYK2,HMOX1,CCR5,EGFR,MAPK1,MMP3,F2,NTRK1,SRC |

| IA16 | Glaucocalyxin D | MAPK14,PTGS2,CYP2C9,AR,PIK3CB,PIK3CD,PIK3CG,PIK3CA,MAPK8,JAK1,MDM2,KDR,AKT1,PARP1,ALOX5,MMP9,MMP1,TLR9,TYK2,CCR5,EGFR,MAPK1,MMP3,F2,CTRC,CXCR2,MET,CD38 |
| --- | --- | --- |
| IA17 | Isopimara-7,15-dien-3-one | PTGS1 |
| IA18 | Isorhamnetin | TERT,PIK3CG,KDR,AKT1,MMP12,PARP1,ALOX5,MMP9,MMP2,MPO,EGFR,MMP3,F2,MET,CD38,SRC,SYK,MCL1,PLA2G1B,XDH,AHR,TTR |
| IA19 | Ladanein | PTGS2,AR,NOS2,TERT,PIK3CG,KDR,MMP12,PARP1,ALOX5,MMP9,MMP2,EGFR,MMP3,MET,CD38,SRC,SYK,MCL1,PLA2G1B,XDH,AHR,TTR,CFTR,ODC1 |
| IA20 | Lambertic acid | AR,PPARG,ATP12A,MDM2,KDR,MMP3,SRC,CNR1,TP53 |
| IA21 | Lariciresinol | PDE5A,ALOX5,MCL1 |
| IA22 | Lasiokaurin | TLR9 |
| IA23 | Leukamenin E | ABL1,MAPK14,PDGFRB,PTGS2,AR,JAK1,KDR,PARP1,TLR9,TYK2,EGFR,F2,CTRC,SRC,PRSS1 |
| IA24 | Maslinic acid | PTGS2,AR,G6PD,NOS2,PPARG,TERT,MDM2,ALOX5,MMP1,MMP2,MMP3,PRKCH,PTPN11,ACP1,PLA2G1B,CNR1 |
| IA25 | Methyl rosmarinate | MAPK14,TERT,PTGS1,MTOR,STAT3,ERBB2,MMP12,PARP1,MMP9,MMP1,MMP2,EGFR,MAPK1,MMP3,MET,ADAM17,SYK,HDAC6,MCL1,BCL2,TTR,BCL2L1,BRAF,SERPINE1,NFE2L2,HDAC2 |

| IA26 | Nervosanin B | STAT3,TLR9 |
| --- | --- | --- |
| IA27 | Oridonin | STAT3,TLR9 |
| IA28 | Pedalitin | PTGS2,TERT,KDR,MMP12,PARP1,ALOX5,MMP9,MMP2,MPO,EGFR,MMP3,F2,MET,CD38,SRC,SYK,BCL2,PLA2G1B,XDH,AHR,TTR,CFTR,ODC1 |
| C | Quercetin | TERT,PIK3CG,KDR,AKT1,MMP12,PARP1,ALOX5,MMP9,MMP2,MPO,EGFR,MMP3,F2,MET,CD38,SRC,SYK,PLA2G1B,XDH,AHR,TTR |
| IA29 | Rabdosin B | MAPK14,PTGS2,AR,TERT,PIK3CA,MTOR,JAK1,IL6ST,MDM2,PARP1,MMP1,TLR9,CCR5,MAPK1,MMP3,F2,CTRC,CXCR2,NTRK1,SRC,SYK,HDAC6 |
| IA30 | Rabdoumbrosanin | MAPK14,PTGS2,AR,NOS2,PTGS1,ITGAL,JAK1,MDM2,PARP1,ALOX5,MMP1,TYK2,CHRM3,HMOX1,EGFR,IL6,MAPK1,MMP3,PRKCH,PTPN11 |
| IA31 | Rosmarinic acid | PTGS2,NOS2,TERT,PIK3CG,KDR,AKT1,MMP12,PARP1,ALOX5,MMP9,MMP2,MPO,EGFR,MMP3,F2,MET,SRC,SYK,MCL1,PLA2G1B,XDH,AHR,CYP1A2 |
| IA32 | Sesamin | ALOX5,HIF1A,MCL1 |
| IA33 | Stearic acid | PRKCA,MAPK14,CASP3,CASP1,AR,JAK1,SELE,TYK2,HDAC6,VCAM1,MEN1,ICAM1,HPGD |
| IA34 | Umbrosin A | MAPK14,PTGS2,AR,JAK1,KDR,TYK2,MPO,MAPK1 |
| FA1 | 3,3',4',5,6,7,8-heptamethoxyflavone | PDE5A,PDGFRB,PTGS2,NOS2,TERT,PIK3CG,KDR,AKT1,PARP1,ALOX5,MMP9,MMP2,EGFR,MMP3,F2,MET,SRC,SYK,MCL1,PLA2G1B,XDH,AHR,ODC1 |
| FA2 | 3',4',7,8-Tetramethoxyflavone | PTGS2,NOS2,TERT,PARP1,ALOX5 |

| FA3 | 1. Hydroxy-4- 2. methoxybenzoic acid | MMP9,MMP1,MMP2,MMP8,TTR,SERPINE1,NGFR |
| --- | --- | --- |
| FA4 | 4',5,7,8-tetramethoxyflavone | PTGS2,NOS2,TERT,ALOX5,SYK,MCL1 |
| FA5 | 5,6,7,4'-tetramethoxyflavone | PTGS2,AR,NOS2,TERT,PARP1,ALOX5,SYK,CFTR |
| FA6 | 5,7,4-trimethoxyflavone | ABL1,PTGS2,AR,NOS2,PPARG,TERT,PIK3CG,KDR,AKT1,MMP12,PARP1,ALOX5,MMP9,MMP2,EGFR,F2,MET,CD38,SRC,SYK,MCL1,XDH,TTR,CFTR,ODC1,ALDH2,CYP1A1 |
| FA7 | 5-demethylnobiletin | PTGS2,NOS2,TERT,PIK3CG,KDR,AKT1,MMP12,PARP1,ALOX5,MMP9,MMP2,MPO,EGFR,MMP3,F2,MET,CD38,SRC,SYK,MCL1,PLA2G1B,XDH,AHR,TTR,ODC1 |
| FA8 | Abscisic acid | PRKCA,NOS2,PPARG,ITGAL,CTNNB1,PARP1,ALOX5,ICAM1,ITGB2,ACE |
| FA9 | Acacetin | PTGS2,TERT,MMP12,PARP1,ALOX5,MMP9,MMP2,CD38,SYK,MCL1,XDH,AHR,TTR,CFTR |
| FA10 | Apigenin | PTGS2,AR,TERT,KDR,AKT1,MMP12,PARP1,ALOX5,MMP9,MMP2,MPO,EGFR,MMP3,F2,MET,CD38,SRC,SYK,XDH,AHR,TTR,CFTR |
| FA11 | Auranetin | PDE5A,PDGFRB,PTGS2,NOS2,TERT,PIK3CG,MAPK8,ADORA2B,KDR,AKT1,ALOX5,MMP9,MMP2,EGFR,MMP3,F2,MET,SRC,SYK,MCL1,XDH,ODC1,CYP1A2,CYP1A1 |
| FA12 | Auraptene | PRKCA,ABL1,CRHR1,PDE5A,NOS2,PIK3CA,JAK1,SELE,PARP1,MMP9,MMP1,MMP2,CHRM3,CXCR2,SYK,CCND1,ICAM1,PRKCB,TLR4,NOD1,ATP4B,ATP4A |
| FA13 | Byakangelicol | OXTR,ABL1,CRHR1,PDE5A,MAPK14,CASP3,PTGS1,PIK3CB,PIK3CD,PIK3CG,PIK3CA,MAPK8,JAK1,ADORA2B,PARP1,TYK2,CXCR2,CD38,SYK,ELANE,CCND1,CCKBR,DNMT3A,RPS6KB1,NFKBIA,NQO1 |

| FA14 | Chrysoeriol | PTGS2,TERT,KDR,AKT1,MMP12,PARP1,ALOX5,MMP9,MMP2,MPO,EGFR,MMP3,F2,MET,CD38,SRC,SYK,MCL1,PLA2G1B,XDH,TTR,CFTR |
| --- | --- | --- |
| FA15 | Deacetylnomilin | MAPK14,AR,TERT,PIK3CB,PIK3CD,PIK3CG,PIK3CA,MAPK8,MTOR,JAK1,ADORA2B,KDR,AKT1,PARP1,ALOX5,MMP9,MMP1,CHRM3,MMP8,EGFR,MAPK1,MMP3,NTRK1,ADAM17,SYK,CCND1,TNF,MMP7 |
| FA16 | Demethylnobiletin | PTGS2,NOS2,TERT,PIK3CG,KDR,AKT1,MMP12,PARP1,ALOX5,MMP9,MMP2,MPO,EGFR,MMP3,F2,MET,CD38,SRC,SYK,MCL1,PLA2G1B,XDH,AHR,TTR,ODC1 |
| FA17 | Diosmetin | PTGS2,TERT,KDR,AKT1,MMP12,PARP1,ALOX5,MMP9,MMP2,MPO,EGFR,MMP3,F2,MET,CD38,SRC,SYK,MCL1,PLA2G1B,XDH,TTR,CFTR |
| FA18 | Epoxyaurapten | PDE5A,MAPK14,PIK3CB,PIK3CD,PIK3CG,PIK3CA,MTOR,ERBB2,ADORA2B,MDM2,KDR,MMP1,EGFR,MET,HDAC2 |
| FA19 | Epoxybergamottin | ABL1,CRHR1,MAPK14,CASP3,PIK3CB,PIK3CD,PIK3CG,PIK3CA,MAPK8,MTOR,JAK1,ADORA2B,AKT1,MMP12,PARP1,TYK2,EGFR,MAPK1,CD38,NTRK1,SYK,HDAC6,CNR1,HDAC2,ATM |
| FA20 | Eriodictyol | PTGS1 |
| FA21 | Hesperitin | PPARG,TERT,PTGS1,KDR,MMP12,PARP1,MMP9,MMP2,MET,NTRK1,SRC,BCL2,PLA2G1B,ODC1,SERPINE1,DNMT1,STAT1 |
| FA22 | Homoeriodictyol | TERT,PTGS1,KDR,MMP12,MMP9,MMP2,SRC,PLA2G1B,ODC1,SERPINE1,DNMT1,STAT1 |

| FA23 | Limonin | OXTR,MAPK14,PDGFRB,CASP3,CASP1,AR,PIK3CB,PIK3CD,PIK3CA,MTOR,ADORA2B,MDM2,KDR,AKT1,MMP9,MMP1,CHRM3,MAPK1,F2 |
| --- | --- | --- |
| FA24 | Luteolin | PTGS2,AR,TERT,KDR,AKT1,MMP12,PARP1,ALOX5,MMP9,MMP2,MPO,EGFR,MMP3,F2,MET,CD38,SRC,SYK,PLA2G1B,XDH,AHR,TTR,CFTR |
| FA25 | Marmin acetonide | CRHR1,MAPK14,CASP3,CASP1,PIK3CG,MDM2,KDR,ALOX5,MMP1,TLR9,HMOX1,MAPK1,CXCR2,NTRK1,CASP8,MCL1,CNR1,BCL2L1,BRAF,HPGD,SNCA |
| FA26 | Meranzin | MAPK14,PIK3CB,PIK3CD,PIK3CG,PIK3CA,MAPK8,MTOR,ADORA2B,EGFR,NTRK1,TGFBR1,HDAC2 |
| FA27 | Methoxsalen | CYP1A2 |
| FA28 | Narcotine | ABL1,PDE5A,MAPK14,PTGS2,CYP2C9,CYP2C19,NOS2,TERT,PIK3CB,PIK3CD,PIK3CG,PIK3CA,MAPK8,MTOR,ERBB2,JAK1,MMP9,MMP1,MMP2,TYK2,EGFR,MAPK1,NTRK1,ADAM17,SYK,TGFBR1,CCND1,HDAC2,ATM |
| FA29 | Naringenin | CYP2C9,PPARG,PTGS1,PIK3CB,PIK3CA,CYP3A4,KDR,MMP12,MMP9,MMP2,MMP3,MET,SRC,SYK,BCL2,PLA2G1B,BCL2L1,SERPINE1,F3,VEGFA,SNCA |
| FA30 | Natsudaidain | PTGS2,NOS2,TERT,PIK3CG,KDR,AKT1,ALOX5,MMP9,MMP2,MPO,EGFR,MMP3,F2,MET,SRC,SYK,MCL1,PLA2G1B,XDH,AHR |
| FA31 | Nobiletin | ALOX5 |

| FA32 | Nomilin | MAPK14,CYP2C9,CASP1,CYP2C19,PIK3CA,MAPK8,MTOR,ITGAL,STAT3,KDR,PARP1,MMP1,MMP2,CHRM3,EGFR,MAPK1,CXCR2,NTRK1,CASP8,TNF,CNR1,ICAM1,ITGB2,CASR |
| --- | --- | --- |
| FA33 | Obacunoic acid | PDE5A,CYP2C9,CASP3,CASP1,PPARG,MAPK8,MDM2,KDR,MMP12,MMP9,MMP1,MMP2,MMP8,MMP3,ADAM17,CASP8,TNF,CCKBR,ACE,ACE2,GZMB,CCKAR |
| FA34 | Obacunone | OXTR,MAPK14,CYP2C9,CASP3,AR,CYP2C19,MAPK8,MDM2,KDR,PARP1,CHRM3,MIF,MAPK1,CXCR2,SRC,CNR1 |
| FA35 | Phellopterin | CRHR1,PDE5A,MAPK14,PIK3CB,PIK3CD,PIK3CG,PIK3CA,MAPK8,MTOR,JAK1,ADORA2B,PARP1,TYK2,CD38,CCND1,CYP1A2,CCKBR,DNMT3A,RPS6KB1 |
| FA36 | Phenylalanine | EGFR,TH |
| FA37 | Sinensetin | PTGS2,NOS2,TERT,PIK3CG,KDR,MMP12,PARP1,ALOX5,MMP9,MMP2,MPO,EGFR,F2,MET,CD38,SRC,SYK,PLA2G1B,XDH,AHR,TTR,CFTR,ODC1 |
| FA38 | Tangeretin | PTGS2,AR,NOS2,TERT,PIK3CG,JAK1,KDR,AKT1,PARP1,ALOX5,MMP9,MMP2,MPO,EGFR,MMP3,F2,MET,SRC,SYK,MCL1,XDH,AHR,ODC1,CYP1A2,CYP1A1,NTRK2 |
| FA39 | Tyrosine | EGFR,TH |
| FA40 | Umbelliferone | PDGFRB,KDR,AKT1,PARP1,EGFR,MIF,MET,CCND1,XDH,CYP1A2,ALDH2,SNCA,HSPA1A,NFKB1 |
| FA41 | Isosakuranetin | PTGS1,PLA2G1B |
